# σ–σ^*^ conjugation Across Si─O─Si Bonds

**DOI:** 10.1002/marc.202500081

**Published:** 2025-03-12

**Authors:** Zijing Zhang, Cecilia Pilon, Hana Kaehr, Pimjai Pimbaotham, Siriporn Jungsuttiwong, Richard M. Laine

**Affiliations:** ^1^ Dept. of Materials Science and Engineering University of Michigan H.H. Dow, 2300 Hayward St Ann Arbor Michigan 48109‐2136 United States; ^2^ Department of Chemistry University of Michigan 505 S. State St Ann Arbor Michigan 48109‐1055 United States; ^3^ Department of Chemistry and Center of Excellence for Innovation in Chemistry Faculty of Science Ubon Ratchathani University Ubon Ratchathani 34190 Thailand; ^4^ Macromolecular Science and Eng. University of Michigan Ann Arbor MI 48109‐21236 USA

**Keywords:** conjugation across Si─O─Si bonds, DP dependent redshifts in emission, large changes in bond angles on excitation, polysiloxane copolymers, sigma–sigma^*^ conjugation

## Abstract

Polysiloxanes and silsesquioxanes (SQs) are known to be insulating materials. We describe here polysiloxane copolymers where this is not the case. Thus,Me_2_VinylSi─O─SiMe_2_Vinyl/Br‐Ar‐Br copolymers exhibit conjugation via Si─O─Si bonds contrary to the widespread understanding that such linkages must be insulating. Here we describe the synthesis, characterization, and photophysical properties of [‐VinylSiMe_2_OMe_2_SiVinyl‐Ar]x copolymers; Ar = phenyl, terphenyl, stilbene, thiophene, etc. Con‐jugation is evidenced by redshifted emission λ_max_ of copolymers vs model compounds, [(MeO)_2_SiMeVinyl‐Ar‐VinylMeSi(OMe)_2_], electron transfer to F4TCNQ and MW (DP) depend‐ent emission red‐shifts (smaller bandgaps with increasing DP). Theoretical calculations targeting electronic structure, absorbance/emission λ_max_ of model com‐pounds vs oligomers support conjugation via π‐dπ^*^ orbital interactions. In the ground state, model compounds offer Si─O─Si bond angles of ≈110° on average. In the copolymers, bond angles change in the ground state averaging ≈ 140 ° and in the excited state approach 150 ° much closer to planarity, a result of conjugation. Here SiOSi bonds facilitate intersystem charge transfer (ICT) as seen in carbon based polymers. Thus, i.e, ICT in VySiOSiVycoPh likely leads to a much larger Stokes shift (≈115 nm) than in the silane model. Our findings provide the first detailed photophys‐ical studies of conjugation in polysiloxane‐chromophore copolymers.

## Graphical abstract

Polysiloxanes and silicone rubbers are well‐recognized as insulating materials and are used widely in multiple commercial applications ranging from electrical insulation, to heating oil baths, lubricants, to caulking/sealants. Contrary to traditional views, we describe here polysiloxane copolymers that exhibit σ–σ^*^ conjugation which to our knowledge is completely unprecedented!

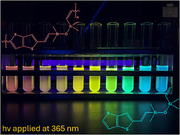

## 1 Introduction

Poly‐siloxanes and ‐silsesquioxanes (SQs) are widely accepted as insulating oils and/or rubber‐like materials and are used extensively in commercial applications where superior insulation and hydrophobicity are desirable. In previous work on functionalized and polymeric SQs, we described the formation of cage‐centered LUMOs that offer 3‐D conjugation with appended functional groups and in T_8,10,12_ cages and co‐polymers linked via divinyl benzene or diethynylbenzene tethers.^[^ ^1^, ^2^, ^3^ ^]^

In efforts to find cage systems where conjugation is not observed, we synthesized and characterized T_8_ cages with corners missing,^[^ ^4^ ^]^ opposing edges open or with [Me_2_Si(O‐)_2_] endcaps (double‐decker, DD).^[^ ^3^ ^]^ Bromination/iodination followed by Heck catalytic cross‐coupling with conjugated moieties provided multiple examples of molecules exhibiting conjugation via several spectroscopic methods, e.g. redshifted emission λ_max_ versus model compounds. This behavior was explained by the formation of cage‐centered LUMOs enabling 3D conjugation as confirmed by the formation of cage‐centered spherical magnetic fields on exposure to intense laser light coupled with modeling studies.^[^ ^5^, ^6^ ^]^ In continuing efforts, we synthesized DD copolymers end‐capped with [MeVinylSi(O‐)_2_] expecting “insulating” disiloxane units would eliminate LUMO formation and thus conjugation.^[^ ^7^ ^]^ Surprisingly, conjugation was again seen as evidenced by emission λ_max_ redshifts, the ability to donate electrons to F_4_TCNQ, and in terpolymers with two different aromatic units such that emission redshift λ_max_ averaged the two homo‐co‐polymers despite the disiloxane linkers.

Thereafter, we synthesized ladder (LL) SQ copolymers with MeVinylSi(O‐)_2_ endcaps assuming the absence of a cage would eliminate conjugation.^[^ ^8^, ^9^ ^]^ However, these systems with smaller DPs than the DD analogs also show conjugation as evidenced by redshifts for the same co‐monomer greater than for the DD analogs. Thus, cage‐centered LUMOs are not needed for conjugation.

**Figure 1:**
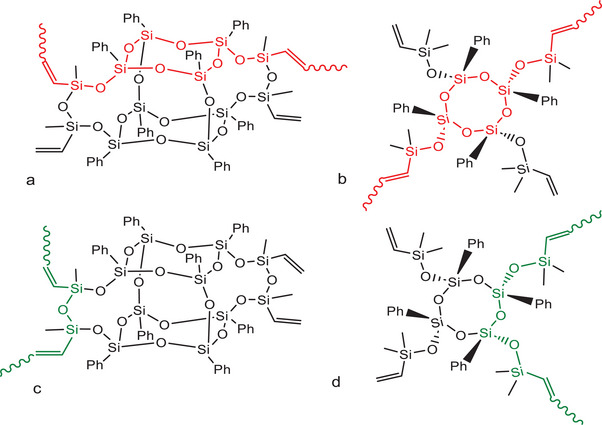
Conjugation follows a trans pathway via the ring a,b) or a *cis* pathway c,d).

In efforts to push the limits of how conjugation arises in these systems, we synthesized copolymers using extended cage double deckers (XDDs) and half cages (HCs) encapped with two vinylmethyldisiloxane units (Vy_4_XDD, Vy_4_HC) **Figure** **1**.^[^ ^10^, ^11^ ^]^ These compounds enable mapping photophysical property differences versus cage sizes and symmetries compared to the T_8,10,12_ copolymers studied previously.

Again, these systems albeit more complicated than earlier studies with four vinyl units per monomer also show evidence for conjugation as determined by UV–vis redshifts, one and two‐photon absorption behavior, electron donation to F_4_TCNQ to form F_4_TCNQ^−.^, as well as redshifted λ_max_ versus DP,^[^ ^10^, ^11^ ^]^ longer chains give larger redshifts.

One concerning aspect of the XDD and HC studies is that conjugation may occur via either the cis (red) or trans (green) forms or both. A second unresolved aspect concerns modeling. Three previous efforts were unable to identify a reasonable mechanism whereby siloxane end‐capped DD and LL copolymers exhibit conjugation. One possible mechanism via dπ‐pπ conjugation, Figure S1 (Supporting Information), was reported recently.^[^ ^10^ ^]^ However, publications by Fujimoto et al and West et al on hyperconjugation between oxygen lone pairs and *σ* ^*^ Si─C bonds suggests an another explanation.^[^ ^12^, ^13^ ^]^

Consequently, we sought to resolve both issues via the synthesis of ‐vinylMe_2_Si‐O‐SiMe_2_vinyl‐Ar copolymers. We present here evidence that belies the long‐standing idea that polysiloxanes are insulating, demonstrating conjugation in ‐[VySiOSiVy‐Ar‐]_x_ copolymers. We further find that hyper conjugation via σ–σ^*^ conjugation plays a major role in the properties we observe.

## 2 Results and Discussion

### 2.1 Syntheses and Characterization

Model silane compounds and ‐[VySiOSiVy‐Ar‐]_x_ co‐oligomers/polymers were synthesized (**Scheme** **1**) via Heck cross‐coupling and characterized per methods reported previously^[^ ^7^, ^8^ ^]^ and in Tables S1–S3 and Figures S2–S22 (Supporting Information). With the exception of VySiOSiVycoBithio (in **Figure** **2**a) and VySiOSiVycoTerph (in Figure 2b), GPC determined MWs reveal the synthesized copolymers’ DPs of 6–60 comonomer units. Polydispersities (Đ) for all oligomers/polymers are ≈2, as expected for step‐growth polymerization.

**Scheme 1:**
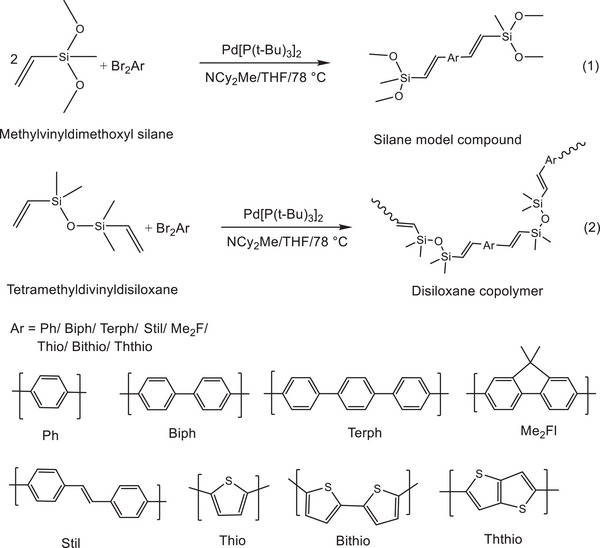
Syntheses of model compounds and typical copolymers‐see experimental for details.

**Figure 2:**
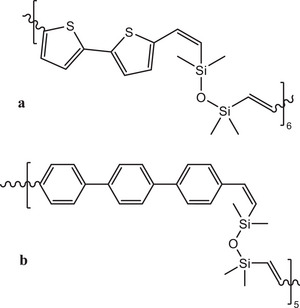
Structure of VySiOSiVycoBithio a) and VySiOSiVycoTerph b).

In particular, VySiOSiVycoBithio oligomers offer DPs ≈6 as seen in other bithiophene SQ copolymers,^[^ ^7^, ^8^ ^]^ as reversible sulfur binding to the Pd catalyst inhibits catalytical activity.^[^ ^14^, ^15^, ^16^, ^17^ ^]^ In contrast, the THF soluble VySiOSiVycoTerph component isolated also offers DPs ≈5 coincident with a THF insoluble but DCM soluble fraction [VySiOSiVycoTerph (ppt)] that offers DPs > 50.

TGA found ceramic yields, Figures S15–S22 and Table S3 (Supporting Information), reveal T_d5%/air_ range 200°–350 °C; lower than calculated, but expected given polysiloxane polymers decompose by backbiting.^[^ ^18^ ^]^

### 2.2 Modeling Photophysical Behavior

**Figure 3:**
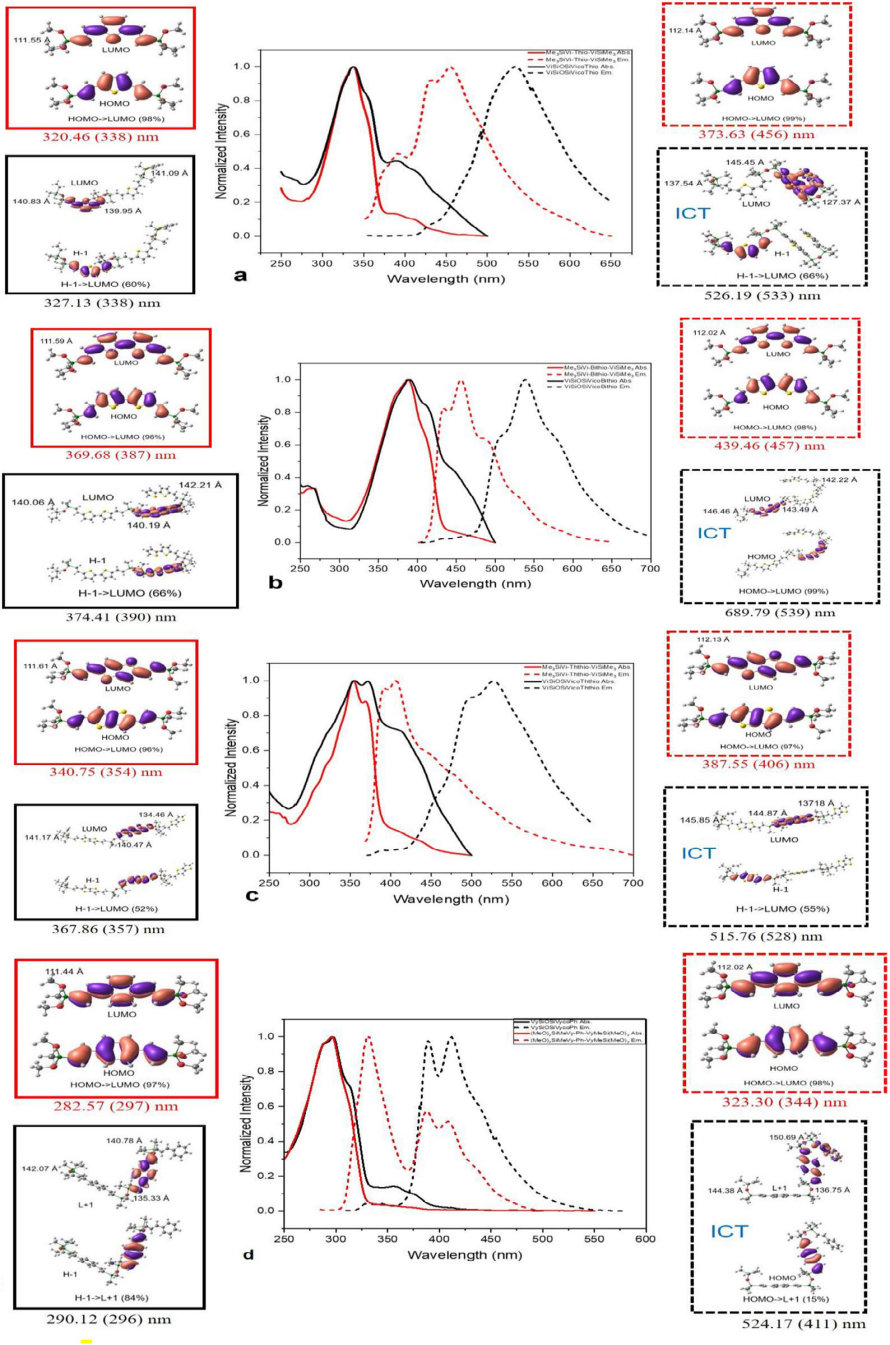
Normalized absorption (solid lines) and emission (dashed lines) and corresponding silane model compound found and calculated spectra and structural conformations for a) VySiOSiVycoThio (black) and (MeO)_2_SiMeVy‐Thio‐VyMeSi(OMe)_2_ (red). b) for VySiOSiVycoBithio (black) and model compound (MeO)_2_SiMeVy‐Bithio‐VyMeSi(OMe)_2_ (red). c) VySiOSiVycoThthio (black) and (MeO)_2_SiMeVy‐Ththio‐VyMeSi(OMe)_2_ (red). d) VySiOSiVycoPh (black) and (MeO)_2_SiMeVy‐Ph‐VyMeSi(OMe)_2_ (red). e–h) in Figure S23 (Supporting Information).

**Figures** **3** and S23 (Supporting Information) present selected Uv–Vis spectra and density functional theory (DFT) calculations elucidating the electronic structure and absorption and emission *λ*_max_ for copolymer and model compounds as recorded in **Table** **1**.^[^ ^8^ ^]^ Geometry optimization was carried out at the B3LYP/6‐31G(d,p) level of theory and the electronic absorption and emission spectra were calculated using time‐dependent DFT (TD‐DFT) calculations at the CAM‐B3LYP/6‐31G(d,p) level. All copolymers display redshifted emission λ_max_ of 40 to 120 nm, and an increased absorption shoulder compared to model compounds, suggesting an extension of conjugation through siloxane bonds.

**Table 1:** Absorption and emission of silane model compounds and copolymers.

	λ_max_ Abs [nm]^a)^	λ_max_ Em [nm]	Stokes Shift [cm^−1^]	Em. λ_max_ redshift vs Silane model [nm]	Φ_F_	Molar absorptivity	DP
Copolymers							
coThio	338	533	10 824	103	0.05 ± 0.01	1.35 ± 0.03	22
coBithio	390	539	7088	82	0.23 ± 0.03	2.66 ± 0.15	6
coThthio	357, 372	528	9072	122	0.18 ± 0.02	4.11 ± 0.10	14
coPh	296	411	9453	79	0.10 ± 0.01	2.21 ± 0.07	63
coBiph	311	408	7645	38	0.27 ± 0.02	2.75 ± 0.005	21
coTerph	317	374, 389	4808	–	0.59 ± 0.07	1.97 ± 0.02	5
coTerph (ppt)	317	457	9664	84	0.46 ± 0.04	–	–
coStil	339, 352	424, 446	5988	37	0.34 ± 0.04	1.70 ± 0.05	11
coMe_2_Fl	336	455, 478	7784	73	0.78 ± 0.09	4.42 ± 0.02	41
Silane model compounds							
‐Thio‐ ^[^ ^8^ ^]^	340	390, 430	6156	–	0.01 ± 0.001	–	1
‐Bithio‐ ^[^ ^8^ ^]^	388	457	3891	–	0.02 ± 0.005	–	1
‐Ththio‐ ^[^ ^8^ ^]^	354	406, 454	3618	–	0.02 ± 0.001	–	1
‐Ph‐ ^[^ ^8^ ^]^	297	332, 387	3550	–	0.01 ± 0.001	–	1
‐Biph‐ ^[^ ^8^ ^]^	313	355, 370	4922	–	0.38 ± 0.03	–	1
‐Terph‐ ^[^ ^8^ ^]^	319	373, 388	4538	–	0.48 ± 0.03	–	1
‐Stil‐ ^[^ ^8^ ^]^	355	391, 409	3719	–	0.36 ± 0.02	–	1
‐Me_2_Fl‐ ^[^ ^8^ ^]^	336	382	3584	–	0.30 ± 0.01	–	1

^a)^ Underlined emission peaks are the highest intensity if multiple peaks. Φ_F_: Quantum yield; Emission spectra were measured with an excitation wavelength corresponding to the absorption λ_max_.

Figure S24 (Supporting Information) shows the geometry‐optimized silane model (MeO)_2_SiMeVy‐Ph‐VyMeSi(OMe)_2_ and VySiOSiVycoPh (Disiloxane copolymer) and calculated absorption (*λ*_max_) values in good agreement with the experimental results. These similar λ_max_ are associated solely with π orbitals localized primarily on the phenyl and adjacent double bonds.

Figure S25 (Supporting Information) shows contributions from the HOMO and LUMO to the (MeO)_2_SiMeVy‐Ph‐VyMeSi(OMe)_2_ model absorption and emission states. In this case, stabilization results in a relatively large energy difference (bandgap) of 6.70 and 5.97 eV for the absorption and emission states, respectively. Here the electrons are confined to ligand conjugation and do not transfer through the O─Si─O bond to the other side involving only a π → π^*^ transition (the HOMO → LUMO). Coincidentally, Si─O─Si bond angles are calculated to be ≈111°.

**Figure 4:**
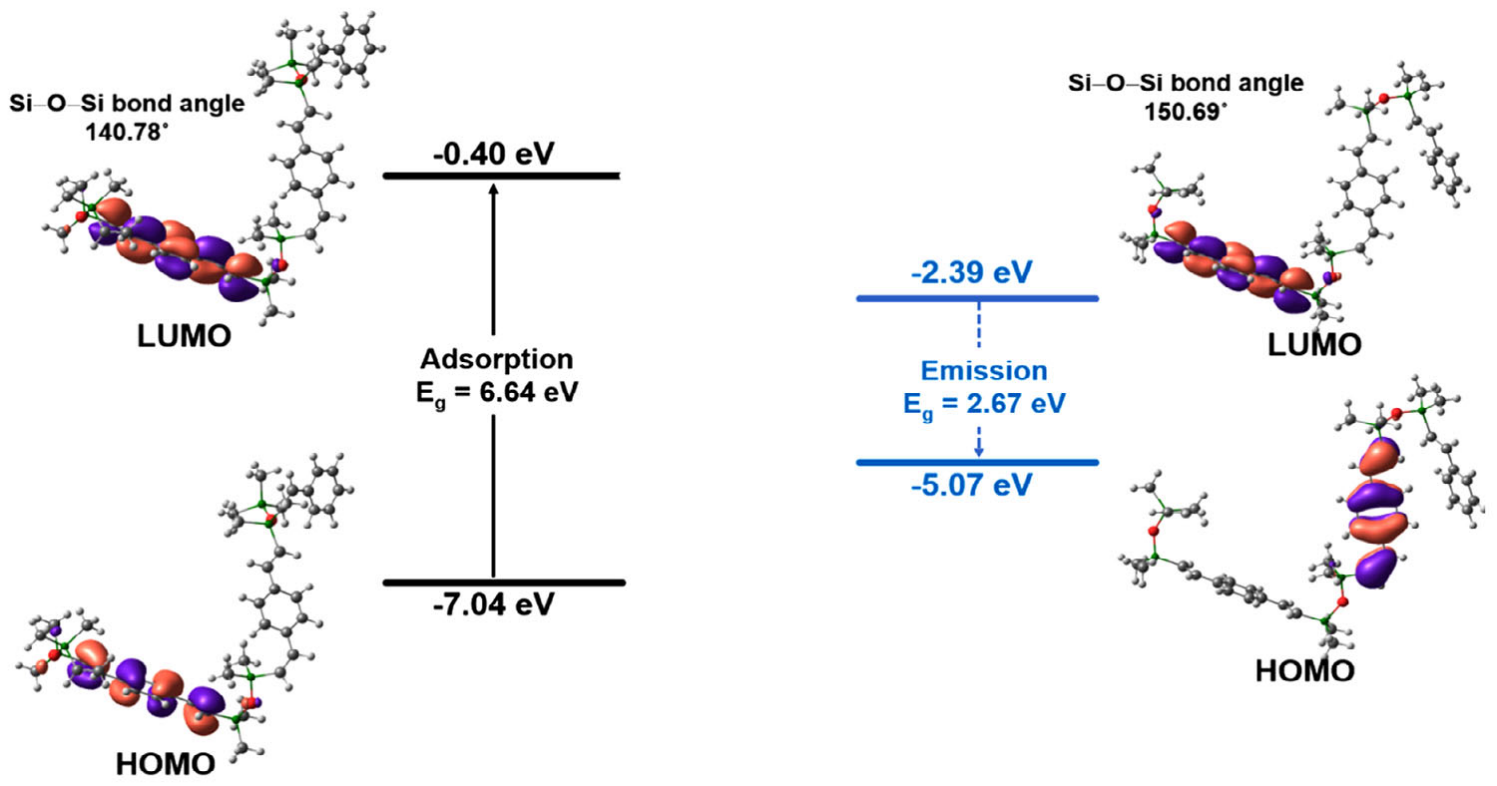
HOMO and LUMO of VySiOSiVycoPh (Disiloxane copolymer) at absorption and emission state.

In contrast, in **Figure** **4** the disiloxane adsorption state indicates a large energy difference, with a Si─O─Si bond angle of 140.78°, while in the emission state, VySiOSiVycoPh exhibits a special LUMO→HOMO electron distribution that delocalizes over Si─O─Si linkage through a single bond *p*(O)σ → σ^*^(Si‐Ar) transition with a Si─O─Si bond angle calculated to be 150.69° indicative of intramolecular charge‐transfer (ICT).^[^ ^21^ ^]^ Conjugation may also occur as noted above between oxygen lone pair *p*‐orbitals, acting as electron donors, and the empty silicon *d*‐orbitals, acting as electron acceptors through p_π_‐d_π_ orbital interactions.^[^ ^19^, ^20^ ^]^ The emission state causes a transformation, resulting in a more planar bond angle in the disiloxane copolymers. As a result, the ability to transfer electrons via Si─O─Si conjugation is enhanced, leading to a small bandgap of 2.67 eV. Similar behavior is found for VySiOSiVycoBithio (bandgap 2.24 eV), Figure S26 (Supporting Information).

These results demonstrate that the Si─O─Si linkages provide bridging conjugation, facilitating charge‐transfer (CT) in a manner similar to carbon conjugation confirming the ICT character of the LUMO→ HOMO state. The ICT process in VySiOSiVycoPh and the other copolymers of Figure 3 offer larger calculated Stokes shifts, Table 1, than silane models as observed experimentally.

### 2.3 Photophysics

As observed, VySiOSiVycoBithio shows an absorption λ_max_ at 390, ≈50 nm redshifted from VySiOSiVycoThio (absorption λ_max_ ≈340 nm). A similar absorption redshift is found for VySiOSiVycoPh, VySiOSiVycoBiph, and VySiOSiVycoTerph. Ar groups with longer conventional π conjugation lengths are likely responsible as seen previously.^[^ ^7^, ^8^, ^10^, ^11^, ^21^ ^]^

The majority of the VySiOSiVy copolymers share the same absorption λ_max_ with their corresponding silane models as seen in earlier SQ‐focused publications,^[^ ^7^, ^8^, ^10^, ^11^, ^21^, ^22^, ^23^ ^]^ however all also exhibit a strong absorption shoulder at longer wavelengths previously suggested to be the more important feature associated with changes in highly efficient emission based on one‐photon and two‐photon excited 2D emission studies.^[^ ^24^ ^]^

This shoulder may arise coincident with the change in the ground state Si─O─Si bond angle versus model compounds from ≈110° to 135°–140°. One explanation is that it arises from a σ→σ^*^ absorption. Furthermore, in the copolymer emission spectra, two sets of emissions are seen; one that corresponds to that of the model compounds and one that can be associated with the polymer. It seems reasonable to suggest that the first set of emissions correspond to isolated segments of the copolymer perhaps separated by defects^[^ ^25^, ^26^ ^]^ that are the equivalent to the model compounds and segments where conjugation occurs over multiple units. The terphenyl insoluble copolymer is perhaps the best example of this, Figure S23f (Supporting Information).

Progressive emission redshifts ranging from 40 to 120 nm are seen moving from silane model compounds to the VySiOSiVy copolymers as the conjugation lengths increase (see following DP‐related studies) reducing the bandgap with detailed photophysical data shown in Table 1 and visualized in Figure 3a–d and Figures S23f,g (Supporting Information). One exception is that the VySiOSiVycoTerph worked up by reprecipitation, which shows a similar absorption λ_max_ and only slightly redshifted emission λ_max_ compared to its model compound because of its small DP. However, the THF insoluble high DP fraction, VySiOSiVycoTerph (ppt) dissolves readily in DCM and exhibits a very large redshifted emission λ_max_ compared to the THF soluble VySiOSiVycoTerph and model compounds which continue to support extended conjugation through siloxane units.

### 2.4 Further proofs of Conjugation, Degree of Polymerization, and Charge‐Transfer Studies

A well‐recognized mechanism for demonstrating conjugation in polymeric systems is to show that as chain length (DP) increases bandgap decreases often evidenced by redshifts in absorption and emission. To further probe structure‐property relationships, column chromatography of VySiOSiVycoStil and VySiOSiVycoBiph allowed the separation of oligomers with different DPs.

Figures S27–S32 (Supporting Information) present fluorescence of the column chromatographic eluates (365 nm), GPC DP analyses, and absorption and emission data irradiation. Colors transition from deep blue to greenish blue as DP increases. Photophysical data are summarized in Table S4 and S5 (Supporting Information).

A more detailed set of studies was undertaken for the VySiOSiVycoMe_2_Fl system versus DP as presented in Figures S33 and Table S6 (Supporting Information). In isolated samples, all the absorption λ_max_ are at ≈336 nm albeit a shoulder develops at ≈400 nm. However, as DP increases from 3 to 13, the peak at 313 nm decreases in intensity and two peaks emerge at 389 and 409 nm, indicating extended conjugation and a decreasing optical bandgap. Figure S33e (Supporting Information) visualizes the changes in the normalized absorption intensities at 313, 389, and 409 nm versus DP. This phenomenon is commonly seen in traditional conjugated organic polymers but is less evident in SQ copolymers and now in polysiloxane copolymers.^[^ ^7^, ^10^, ^11^ ^]^ Notably, as DP increases, a significant emission λ_max_ shifts to 448 nm whereas the 356 nm peak decreases, Figure S33d,f (Supporting Information).

Note that efforts to measure excited state lifetimes, as done previously,^[^ ^11^ ^]^ were unsuccessful because the lifetimes were short enough to fall within the rise time of the interrogating laser.

### 2.5 Charge‐Transfer Studies

To better understand “conjugation” in disiloxane‐derived polymers, a series of mixtures of VySiOSiVy copolymers with F_4_TCNQ was prepared using the mixed solution method.^[^ ^27^, ^28^ ^]^ To map the photophysical properties of the current copolymers, the photophysics of Vy_4_XDD, Vy_4_HC, and VySiOSiVy copolymers are compared.

The emergence of flat panel displays and semiconducting, molecularly doped organic semiconductors (OSCs), including conjugated molecules and polymers, has spurred significant research into understanding charge‐transfer within these materials.^[^ ^29^, ^30^ ^]^ One method of assessing the existence of conjugation in a previously uncharacterized polymer is to determine if conjugation generates a material with the potential to transfer charge to a well‐known electron acceptor, e.g. F_4_TCNQ. We have used this method previously to characterize our novel polymer systems^[^ ^7^, ^9^ ^]^ finding integer charge‐transfer (ICT) between F_4_TCNQ and DD‐ LL‐, HC‐ and XDD‐copolymers.

Here similar ICT was seen mixing 50 mol.% F_4_TCNQ with VySiOSiVy‐co BiPh/Terph/Stil in DCM solutions resulting in obvious color changes from the original no color/yellowish green to dark green for all VySiOSiVy copolymer solutions as seen in Figures S34 and S35. (Supporting Information)

Figure S35 (Supporting Information) presents the absorption spectra for these three VySiOSiVy copolymers with peaks for F_4_TCNQ^−^ at ≈760 and 860 nm corresponding to the D_0_→D_1_ transition.^[^ ^31^ ^]^ VySiOSiVycoTerph shows the strongest F_4_TCNQ^−.^ absorption peaks and is the darkest green in Figure S35 (Supporting Information).

A second method of assessing ICT in such systems is to examine the νC≡N in FTIRs of the mixtures. All the Figure S37 (Supporting Information) copolymer FTIRs for F_4_TCNQ doped materials show characteristic νC≡N peak shifts from 2227 to 2194 cm^−1^ indicative of ICT^[^ ^27^ ^]^ as summarized in Table S7 (Supporting Information) which is consistent with the results from absorption spectrum.

Based on previous research, we can use the above CT data to estimate energy levels for polymers that interact with F_4_TCNQ. When the HOMO of OSC is higher in energy than the dopant's LUMO, ICT will occur.^[^ ^27^ ^]^ Since all VySiOSiVy coBiph, coTerph, coStil polymers exhibit ICT behavior with F_4_TCNQ, with a reported LUMO of −5.2 eV, it is reasonable to suggest that these polymers’ HOMOs should be greater than −5.2 eV.

### 2.6 Mapping Vy_4_XDD, Vy_4_HC and VySiOSiVy Copolymer Structure‐Photophysical Properties

The successful syntheses of Vy_4_XDD, Vy_4_HC, and VySiOSiVycopolymers (Scheme S2, Supporting Information), which share the same aromatic moieties but differ in their SQ/siloxane linkers, enabled us to map their photophysical properties.^[^ ^10^, ^11^ ^]^ Figure S38 (Supporting Information) records both Φ_F_ a) and molar absorptivity b) for these copolymers as summarized in Tables S8 and S9 (Supporting Information). All VySiOSiVy copolymers, except coThio, exhibit lower Φ_F_ compared to their SQ copolymer analogs. This difference is attributable to greater backbone flexibility for VySiOSiVy copolymers resulting in more rotational and vibrational movement likely promoting nonradiative decay.^[^ ^32^ ^]^

Molar absorptivity values offer molecule‐specific information by indicating the molecule absorption strength, reflecting the fundamental properties of its molecular transitions. It is well‐established that ensuring a more planar structure and extending the conjugation length in a molecule typically increases molar absorptivity. The molar absorptivity of these copolymers tends to increase with the geometry/rigidity of the SQ linker. A similar phenomenon was seen that when the single aromatics attached with T_8_ SQ, molar absorptivity increases compared with non‐cage aromatic molecules.^[^ ^33^ ^]^ One explanation is that the rigid Vy_4_XDD SQ core facilitates the separation of aromatic moieties, thereby preventing aggregation and enhancing unconventional conjugation.

## 3 Conclusions

The results presented here demonstrate contrary to widely held beliefs, conjugation through Si─O─Si bonds appears possible and likely occurs by a combination of dπ‐pπ overlap between Si and O and more importantly, strong *σ*–*σ* ^*^ hyperconjugative interactions. In principle, this changes our understanding of the photophysical behavior of several classes of siloxane compounds; albeit, not traditional polysiloxanes based mostly on PDMS.

A further conclusion that might be suggested is that conjugation in the silsesquioxane cages originally attributed to cage‐centered LUMOs may in fact occur in part or totally via peripheral Si─O─Si pathways which would explain the redshifted emission seen in LL/Vy_4_HC copolymer without the formation of the cage structure.

Because we have observed multiple emission pathways from related compounds as noted in our most recent paper, it now becomes possible to suggest the possibility of two or more forms of conjugation in functionalized silsesquioxanes and polysiloxane copolymers which in turn may suggest new applications and properties for these and related classes of materials.

## Conflict of Interest

The authors declare no conflict of interest.

## Supporting information



Supporting Information

## Data Availability

The data that support the findings of this study are available from the corresponding author upon reasonable request.
