# Supplementary material for: σ–σ* conjugation Across Si─O─Si Bonds
Source: Macromol Rapid Commun. 2025 Mar 12;46(10):2500081. doi: 10.1002/marc.202500081 (PMC12087750; doi:10.1002/marc.202500081)
Supplement: Supplementary file 1 — Supporting Information [file MARC-46-2500081-s001.docx]

**σ-σ* conjugation across Si-O-Si bonds**

Zijing Zhang,^1^ Cecilia Pilon,^2^ Hana Kaehr, ^1^ Pimjai Pimbaotham,^3^ Siriporn Jungsuttiwong,^3^ Richard M. Laine^*1,4^

Contribution from the ^1^Dept. of Materials Science and Engineering; ^2^Department of Chemistry; University of Michigan, Ann Arbor, 48109-1055,

^3^Department of Chemistry and Center of Excellence for Innovation in Chemistry, Faculty of Science, Ubon Ratchathani University, Ubon Ratchathani 34190, Thailand; ^4^Macromolecular Science and Eng. University of Michigan, Ann Arbor, 48109-21236,

[talsdad@umich.edu](mailto:talsdad@umich.edu)

**Supporting Information**

**Experimental section**

**Materials**

All purchased chemicals were used as received unless otherwise indicated. 1,3-divinyltetramethyldisiloxane was purchased from ACROS ORGAMOCS (97% purity). Bis(tri-tert-butylphosphine)palladium(0) (Pd[P(t-Bu)_3_]_2_), N,N-dicyclohexylmethylamine (NCy_2_Me) and 1,4-dibromobenzene were purchased from TCI Chemicals-Japan. 1,4-dibromobenzene, 4,4′-dibromo-1,1′-biphenyl, 4,4″-dibromo-p-terphenyl, 2,7-dibromo-9,9-dimethylfluorene, trans-4,4′-dibromostilbene, 2,5-dibromothiophene, 5,5'-dibromo-2,2'-bithiophene, 2,5-dibromothieno[3,2-b]thiophene were purchased from Sigma Al-drich. Tetrahydrofuran was dried over pre-treated molecular sieves for at least 48h and then distilled under nitrogen from sodium/benzophenone ketyl before use.

**Synthetic Methods**

General procedure for oligomerization of 1,3-divinyltetramethyldisiloxane with ArBr_2_.

To a 50 mL flame-dried Schlenk flask under N_2_, a mixture of 1,3-divinyltetramethyldisiloxane (2 mmol. 382 mg), NCy_2_Me (800 mg, 4 mmol) and Br_2_-Ar (2 mmol) in 24 mL dry THF was added. Further, Pd[P(t-Bu)_3_]_2_ (54 mg, 0.1 mmol) was then added, and the flask connected to a condenser sealed with a rubber septum. The mixture was stirred magnetically at reflux for 2 d, cooled and then centrifuged to separate the solution 1 and precipitate 1. The FTIR results in Figure **S3** (purification process of VySiOSiVycoMe_2_Fl reaction is shown as an example) show precipitate 1 contains mostly the byproduct dicyclohexylmethylaminehydrobromide and solution 1 contains partially bromide salt and targeted product with the proof of peak at 1041 cm^-1^ for ν Si-O-Si. The resulting solution 1 was condensed by rotary evaporation and then precipitated into cold, well-stirred methanol (20 mL) to form precipitate 2. The mixture was centrifuged to collect the precipitate 2. The resulting precipitate 2 was vacuum dried at 60 °C to give a solid or waxy product. The FTIR result of precipitate 2 in Figure **S4** shows successful removal of dicyclohexylmethylaminehydrobromide by decreasing the peaks from 2520 to 2920 cm^-1^.

Removal of Pd Catalyst. To a dry 50 mL Schlenk flask under N_2_ were added the above product dissolved in THF (10 mL) and N-acetyl-L-cysteine (0.1 g) dissolved in THF (1 mL). The solution was stirred magnetically overnight at room temperature and then filtered through 1 cm Celite to remove the insoluble Pd−cysteine complex. Most solvent was removed by rotary evaporation, and the resulting solid was redissolved in a minimal amount of THF and slowly poured into cold, well-stirred methanol (20 mL) to fully precipitate the product. The product was then recovered by filtration and dried under vacuum.

Column chromatographic separation of VySiOSiVy-coStil/Me_2_Fl/Biph oligomeric mixture by DP.

Before the column chromatography, TLC was used to determine the proper solvent combination. Elution with a 2:1 v:v hexane: CH_2_Cl_2_ (DCM) provides good separation of oligomers with obvious DP changes. As the short oligomers washed out, the polarity of the solvent was increased until all DCM. A 20 cm long column was prepared using 50 g silica gel (230-450 mesh) and a 2:1 hexane:DCM solvent system allowing separation of polymer fractions according to chain length. Column chromatography allows clear separation of products as seen under 365 nm illumination.

Mixing F_4_TCNQ with VySiOSiVycopolymers:

To a 2 mL vial, 10 mg solid copolymers in 2 ml DCM was added, then different volumes of F_4_TCNQ/DCM (2 mg/mL) solutions were added slowly on the top of the copolymer solution. Other terpolymer solutions with different molar ratios of F_4_TCNQ dopant were prepared using the same procedure. UV-vis spectrometry of the VySiOSiVy copolymers mixed with F_4_TCNQ in DCM were measured. FTIR of the dried VySiOSiVy copolymers mixed with F_4_TCNQ as solid was measured.

**Analytical methods**

Matrix-Assisted Laser Desorption/Time of flight Spectrometry (MALDI-Tof)

The MALDI-ToF analyses were performed on a Bruker Autoflex Speed MALDI-Tof equipped with a 337 nm nitrogen laser in positive-ion reflection mode using 1,8,9-Trihydroxyanthracene, 97%, as matrix, and AgNO_3_ as ion source. Samples were prepared by mixing solutions of 5 parts matrix (10 mg/mL), 5 parts sample (1 mg/mL in THF), and 1part AgNO_3_ (1 mg/mL in water). The mixture was blotted in three different spots of a steel plate with different concentrations before conducting the analyses under high vacuum.

Fourier Transform Infrared Spectroscopy (FTIR)

The FTIR analyses were carried out using a Nicolet 6700 Spectrometer from Thermo Scientific in transmission mode. Approximately 4 mg of sample were mixed in a mortar with 400 mg of KBr and compressed into a sample holder. The spectra were collected for the mixtures and for a background of pure grinded KBr in the range from 400 to 4000 cm ^-1^.

Thermogravimetric analysis (TGA)

The TGA analyses were performed in a SDT Q600 machine from TA instruments. The analyses were carried out by heating approximately 10 mg of sample from 50 to 900 °C at 10°C per min using aluminum pans, previously cleaned by soaking in acqua regia, dis-tilled water and ethanol. The carrier gas was dry air at 60 ml/min.

Nuclear Magnetic Resonance (NMR)

The analyses were carried out in a VarianM400 operated at 400 MHz (9.4 Tesla) Premium Shielded Magnet. The solvent used was deuterated chloroform containing 100 ppm TMS unless indicated otherwise. The analyses were performed by scanning 24 times in concentrations around 10 mg per mL for proton detection and 50 mg per mL for carbon. Simulations were performed using Chem Draw office suite, licensed by University of Michigan.

Gel permeation chromatography (GPC)

GPC analyses were performed in a Waters 515 GPC with in-line degasser and THF as solvent. The samples were prepared by diluting approx. 2 mg of sample into 1 mL THF and filtering through PVDF filters of 0.22 microns pores. The RI detector registered all the products eluting out of the column for 50 min. The estimation of molar masses is given using internal calibration with PS standard solutions with narrow dispersity.

**Photophysical characterization.**

UV-vis spectrometry.

UV-vis measurements were recorded on a Shimadzu UV-1601 UV-vis transmission spectrometer. Samples were dissolved in DCM and diluted to a concentration (10^−4^-10^−5^ M) where the absorption maximum was <10% for a 1 cm path length.

Photoluminescence spectrometry.

Photoluminescent spectra were recorded on a Fluoromax-2 fluorometer in the required solvent using excitation wavelength at the absorption $\lambda$_max_. Samples from UV-vis spectroscopy were diluted (10^−5^-10^−6^ M) to avoid excimer formation and fluorometer detector saturation.

Photoluminescent quantum yields.

Quantum yields were measured using an integrated sphere. Samples were dissolved in CH_2_Cl_2_ (DCM) and diluted to a concentration (10^−4^-10^−5^ M) where the absorption maximum was <10% for a 1 cm path length. Absorption and emission inside the sphere were determined by comparison to a blank CH_2_Cl_2_ in cuvette (glass only). Each sample was measured three times or more.

Molar extinction coefficients (ε) were calculated as: ε = A/cl.

(Where A= absorbance, c = sample concentration in moles/liter and0 l = length of light path through the sample in cm.) All the samples are dissolved in DCM; the cuvettes used for the absorption test have an inner length of 1 cm.


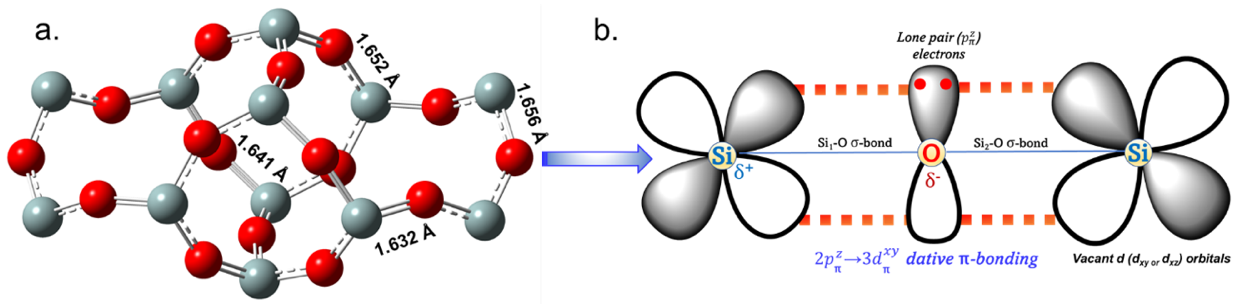


**Figure S1**. Apparent conjugation via dπ-pπ (alternately σ*-π*) interactions.^[1]^

**Physical, structural and thermostable property characterization data.**

**Table S1. Representative copolymer FTIR data for VySiOSiVycoThi**

| **Compound** | **ν** | **Wavenumber (cm^-1^)** | **Intensity** |
| --- | --- | --- | --- |
| **VySiOSiVycoThio** | Ar C=C (bending)  Si-CH_3_  Si-CH_3_  **Si-C**H=CH-  Si-O-Si  Si-CH_3_  Si-**CH=CH**-  Ar C=C  Si-**CH=CH**-  C-H | 740  799  847  974  1050  1251  1410  1512  1593  2957 | Weak, sharp  Strong, sharp  Strong, sharp  Medium, sharp  Strong, broad  Strong, sharp  Weak, sharp  Weak, sharp  Weak, sharp  Weak, broad |

**Table S2**. ^1^ H-NMR peaks of VySiOSiVy copolymers.

| **Copolymers** | **^1^H-NMR peaks (ppm)** |
| --- | --- |
| VySiOSiVycoThio | 1H NMR (401 MHz, cdcl3) δ 7.08 – 6.90 (m, 2H, thiophene), 6.89 – 6.76 (m, 2H, vinyl), 6.14 (dt, J = 18.9, 4.3 Hz, 2H, vinyl), 0.35 – 0.12 (m, 12H, methyl). |
| VySiOSiVycoBithio | ^1^H NMR (401 MHz, cdcl_3_) δ 7.04 – 6.89 (m, 4H, thiophene), 6.85 – 6.70 (m, 2H, vinyl), 6.21 – 5.96 (m, 2H, vinyl), 0.23 (d, *J* = 4.5 Hz, 12H, methyl). |
| VySiOSiVycoThthio | ^1^H NMR (401 MHz, cdcl_3_) δ 7.69 – 7.08 (m, 2H, thiophene), 7.11 – 6.84 (m, 2H,vinyl), 6.25 – 5.70 (m, 2H,viny), 0.44 – 0.10 (m, 12H, methyl). |
| VySiOSiVycoPh | ^1^H NMR (401 MHz, cdcl_3_) δ 7.37 (d, *J* = 4.1 Hz, 4H, phenyl), 7.07 – 6.73 (m, 2H,vinyl), 6.39 (dd, *J* = 28.3, 19.2 Hz, 2H, vinyl), 0.25 (d, *J* = 3.5 Hz, 12H, methyl). |
| VySiOSiVycoBiph | ^1^H NMR (401 MHz, cdcl_3_) δ 7.64 – 7.48 (m, 8H, phenyl), 7.05 (dd, *J* = 19.0, 3.2 Hz, 2H,vinyl), 6.55 (d, *J* = 19.0 Hz, 2H,vinyl), 0.35 (s, 12H, methyl). |
| VySiOSiVycoTerph | ^1^H NMR (401 MHz, cdcl_3_) δ 7.67 – 7.49 (m, 12H, phenyl), 6.99 (dd, *J* = 19.2, 4.3 Hz, 2H,vinyl), 6.48 (ddd, *J* = 19.6, 13.5, 3.5 Hz, 2H, vinyl), 0.31 – 0.24 (m, 12H, methyl). |
| VySiOSiVycoStil | ^1^H NMR (400 MHz, cdcl_3_) δ 7.54 – 7.30 (m, 8H, phenyl), 7.15 – 6.84 (m, 4H, vinyl), 6.45 (d, *J* = 19.1 Hz, 2H, vinyl), 0.26 (s, 12H, methyl). |
| VySiOSiVycoMe_2_Fl | ^1^H NMR (401 MHz, cdcl_3_) δ 7.63 – 7.36 (m, 6H, phenyl), 7.12 – 6.93 (m, 2H, vinyl), 6.51 (dd, *J* = 19.1, 4.8 Hz, 2H, vinyl), 1.45 (d, *J* = 5.3 Hz, 6H, methyl on fluorene), 0.34 – 0.20 (m, 12H, methyl on disiloxane). |

**Table S3**. GPC and TGA data for VySiOSiVy copolymers.

|  | Yield  % | GPC^†^ | | | | TGA | | |
| --- | --- | --- | --- | --- | --- | --- | --- | --- |
|  |  | Mn | Mw | DPI | DP | T_d5%_ ^o^ C | CY  _theorical_ % | CY  _experimental_ % |
| VySiOSiVy |  | 264 | 266 | 1.0 | 1 | - | 65 | - |
| VySiOSiVycoThio | 58 | 3401 | 7532 | 2.2 | 21.7 | 190 | 44 | 19 |
| VySiOSiVycoBithio | 53 | 1339 | 2640 | 2.0 | 6.1 | 249 | 34 | 21 |
| VySiOSiVycoThThio | 44 | 2934 | 5442 | 1.9 | 13.5 | 350 | 37 | 17 |
| VySiOSiVycoPh | 44 | 8088 | 21085 | 2.6 | 62.7 | 355 | 46 | 26 |
| VySiOSiVycoBiph | 78 | 3478 | 8533 | 2.5 | 20.5 | 340 | 36 | 15 |
| VySiOSiVycoTerph | 15 | 1267 | 2568 | 2.0 | 5.2 | 232 | 29 | 14 |
| VySiOSiVycoStil | 47 | 2781 | 4978 | 1.8 | 11.3 | 324 | 33 | 15 |
| VySiOSiVycoMe_2_Fl | 74 | 11498 | 18698 | 1.6 | 40.9 | 343 | 32 | 14 |

DP calculated from Mw.^†^ GPC Mws determined against polystyrene standards.

**
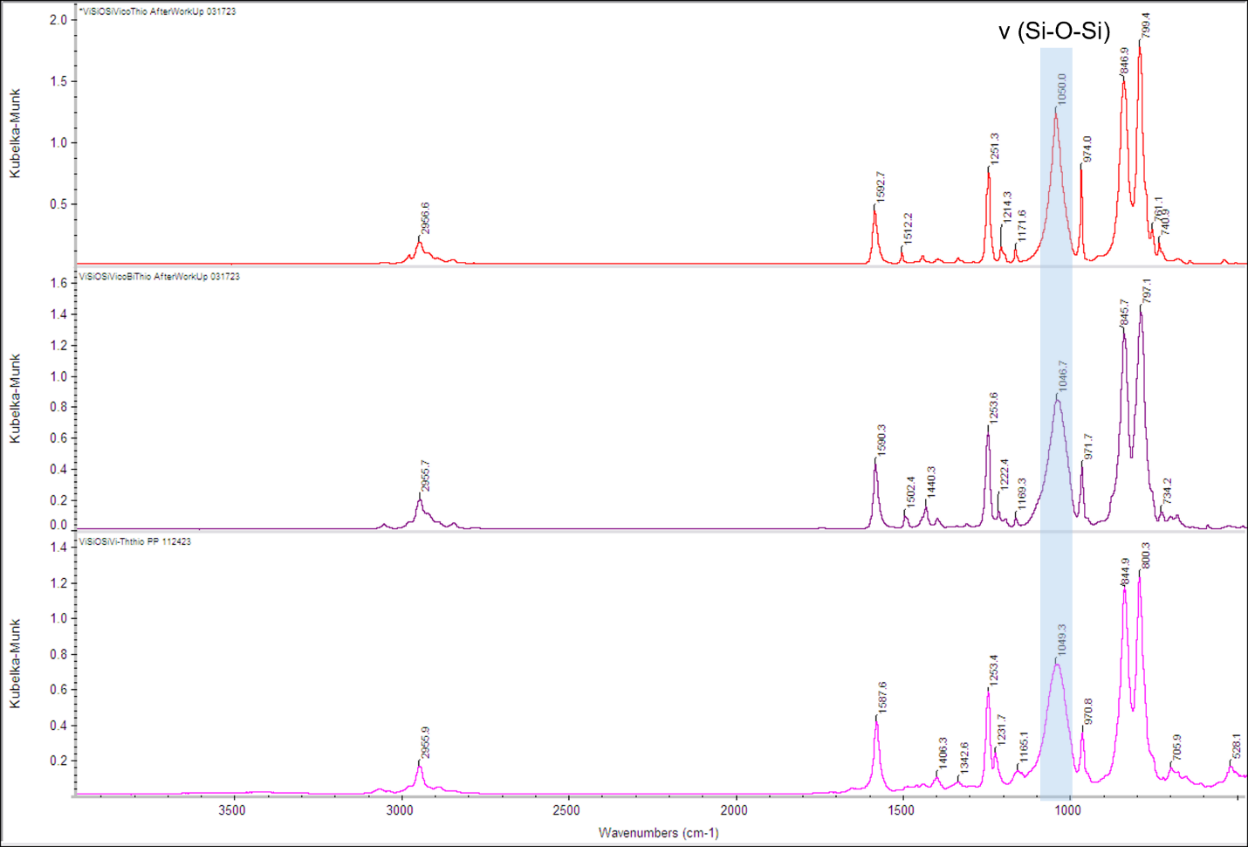
**

**Figure S2**. FTIR of VySiOSiVycopolymer (From top to bottom: coThio/ coBithio/coThthio).

**
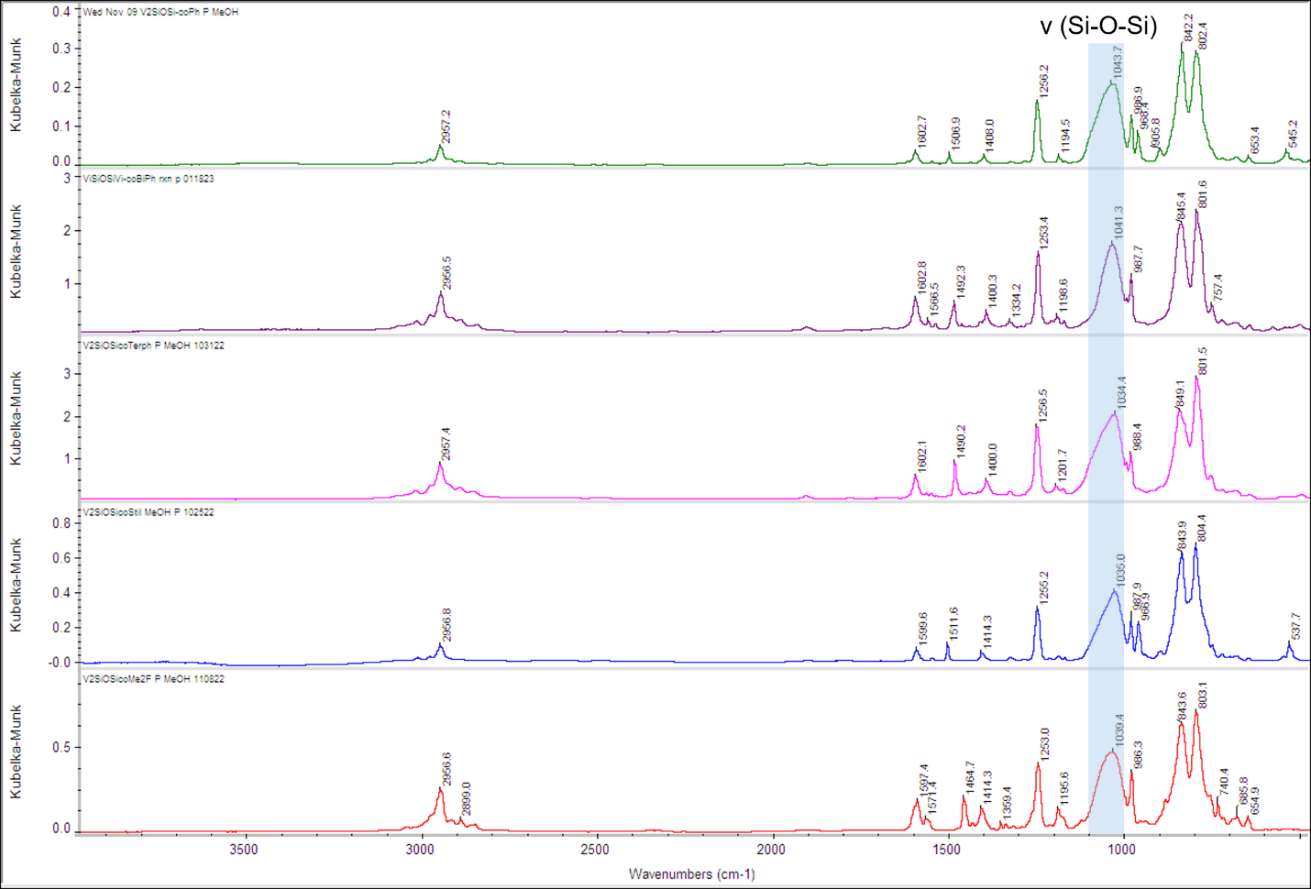
**

**Figure S3**. FTIR of VySiOSiVycopolymer (From top to bottom: coPh/ coBiPh/ coTerph/ coStil/ coMe_2_Fl).

**
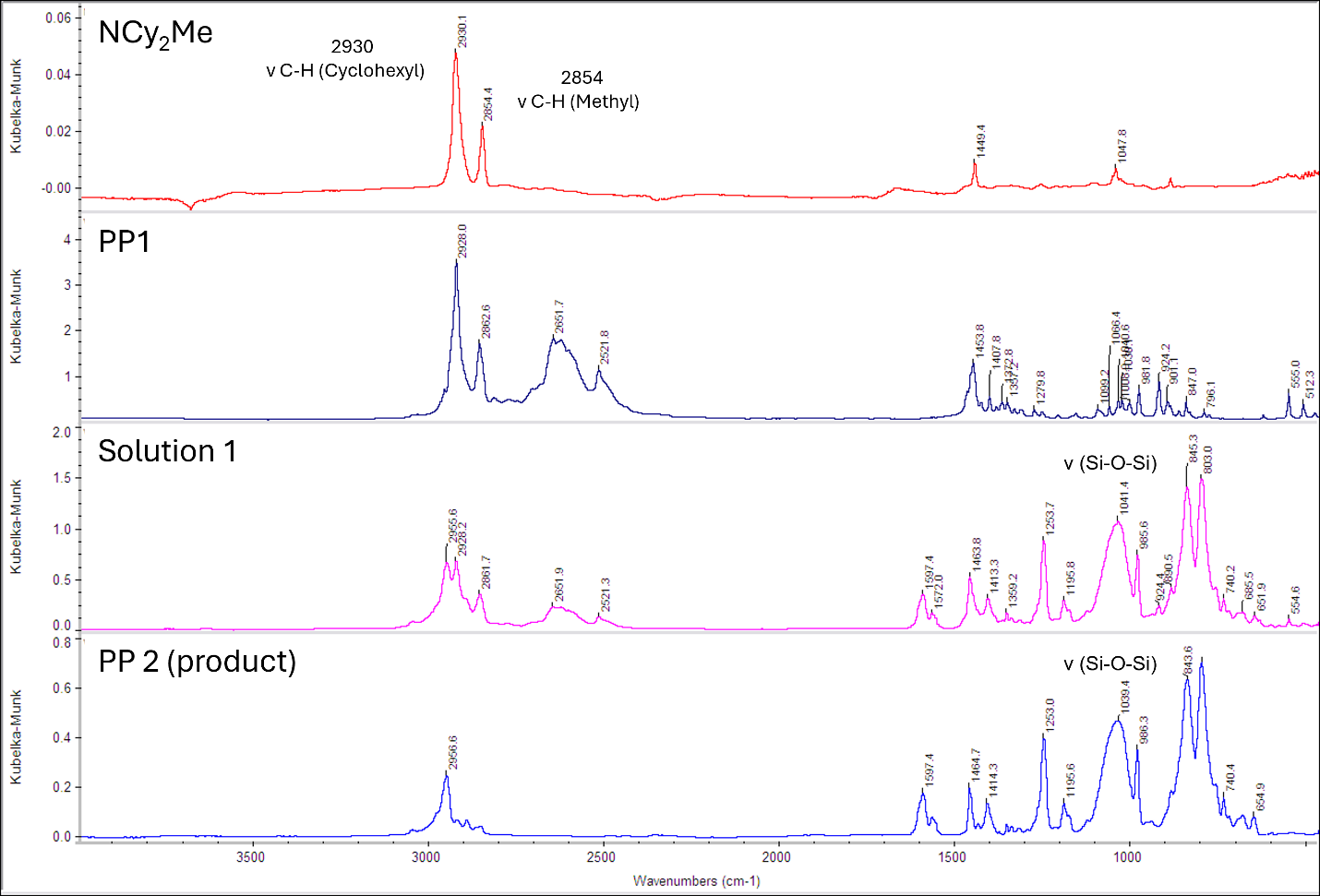
**

**Figure S4**. FTIR of VySiOSiVycoMe_2_Fl purification process.

**
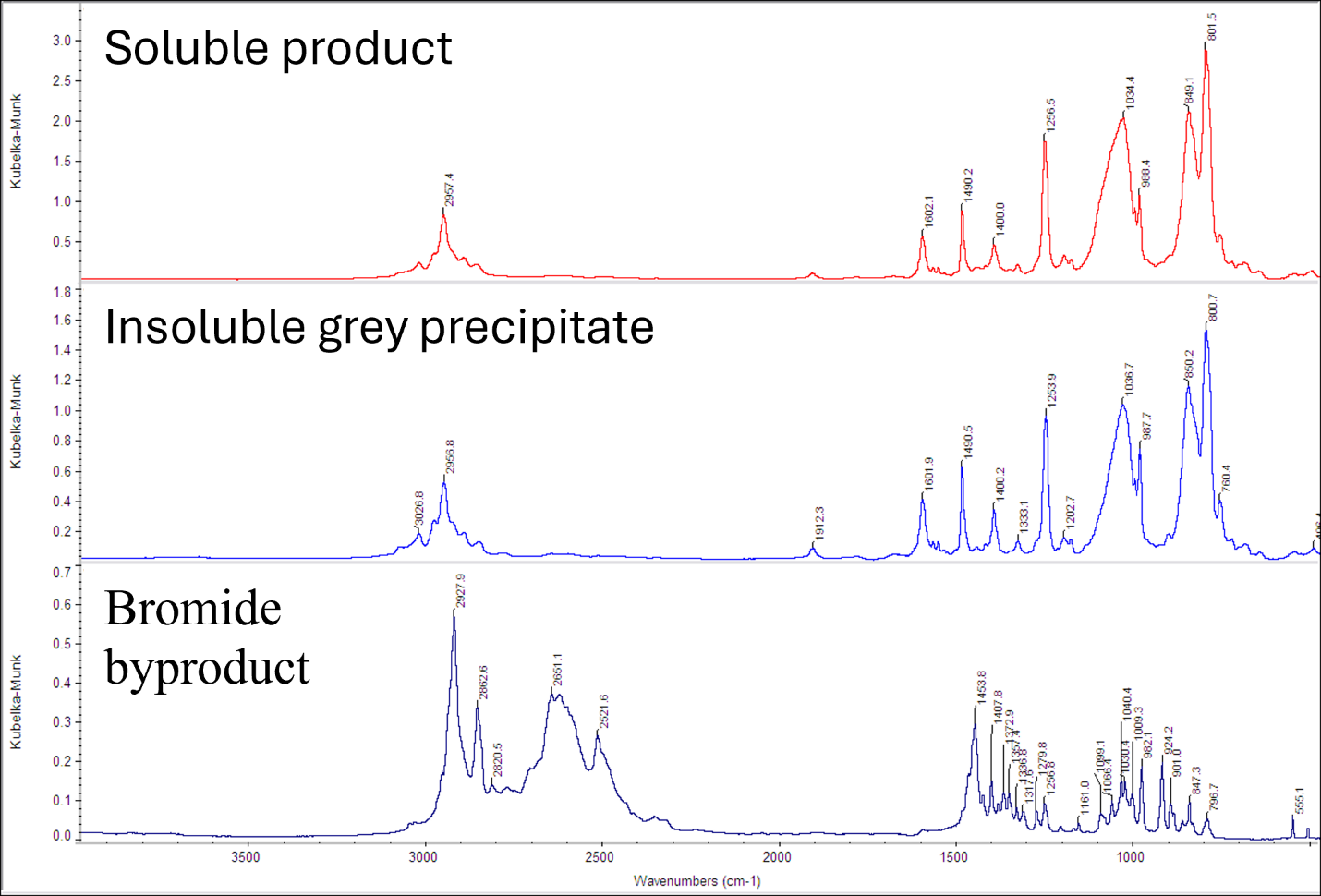
**

**Figure S5**. FTIR of VySiOSiVycoTerph.

**NMR and GPC Data**

**
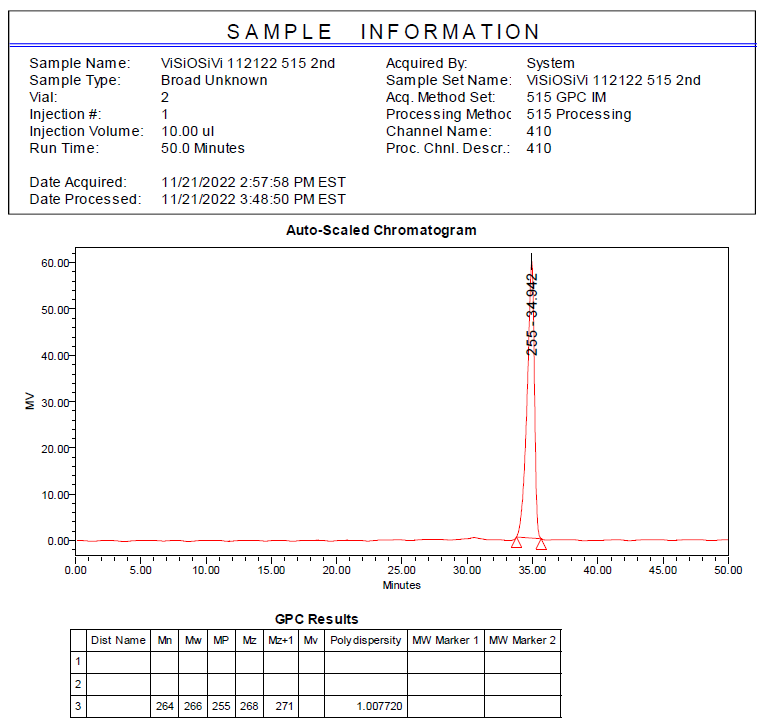
**

**Figure S6**. GPC of the starting divinyltetramethyldisiloxane.


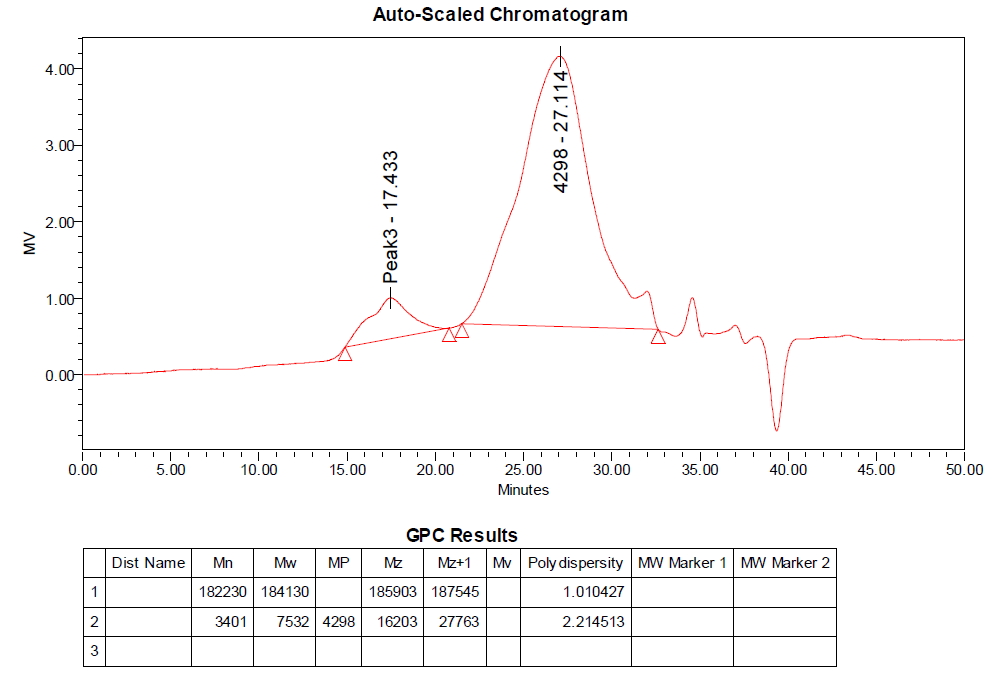


**Figure S7**. GPC of the VySiOSiVycoThio.


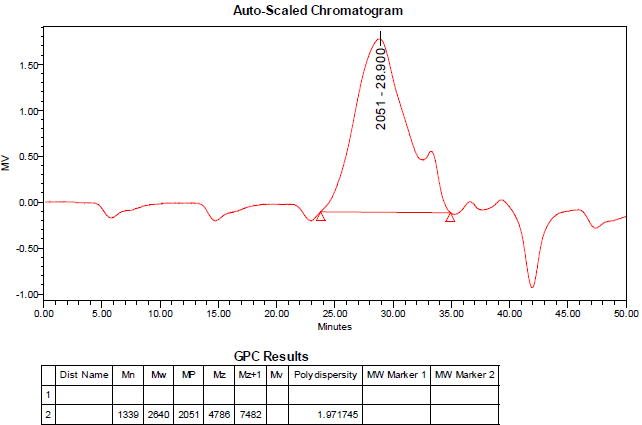


**Figure S8**. GPC of the VySiOSiVycoBithio.


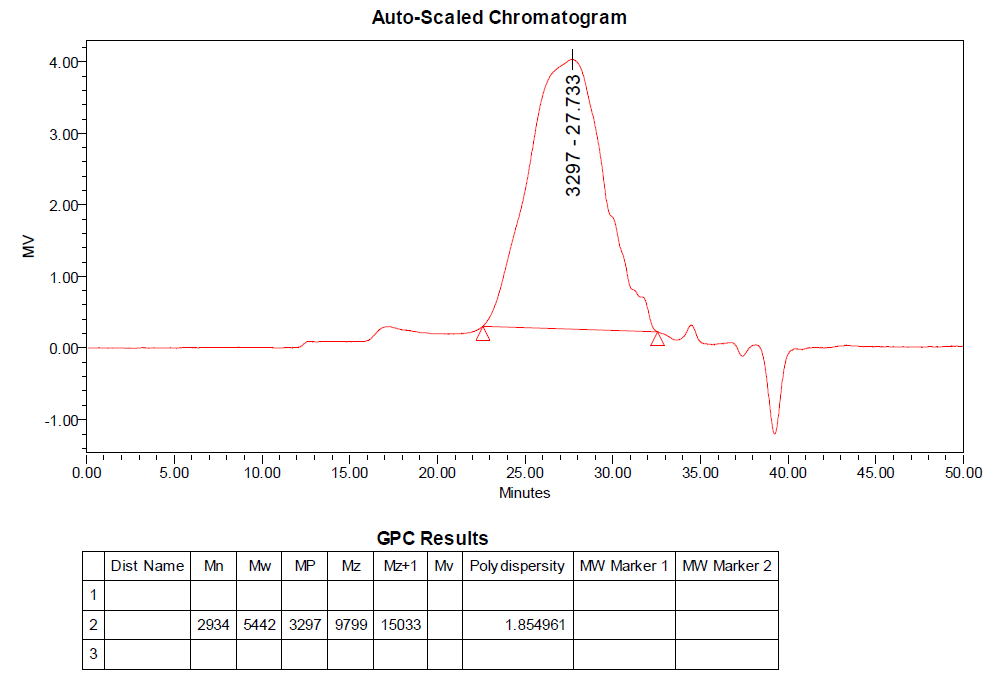


**Figure S9**. GPC of the VySiOSiVycoThThio.


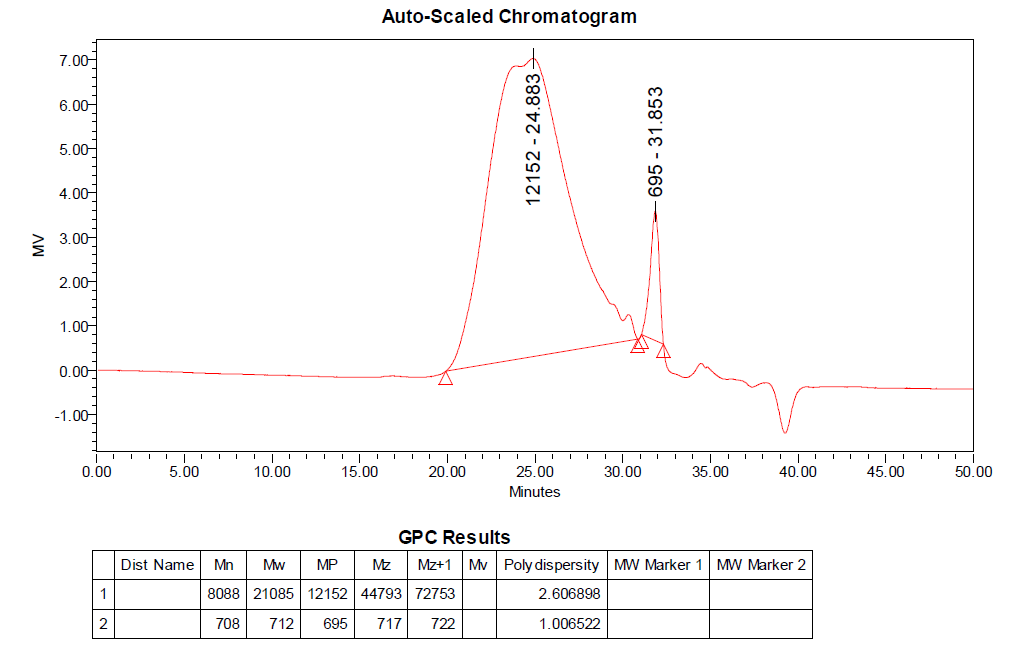


**Figure S10**. GPC of the VySiOSiVycoPh.


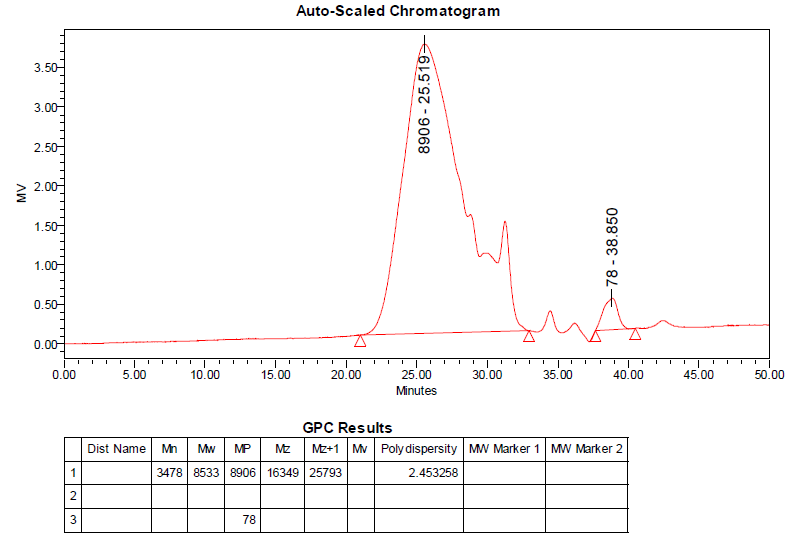


**Figure S11**. GPC of the VySiOSiVycoBiph.


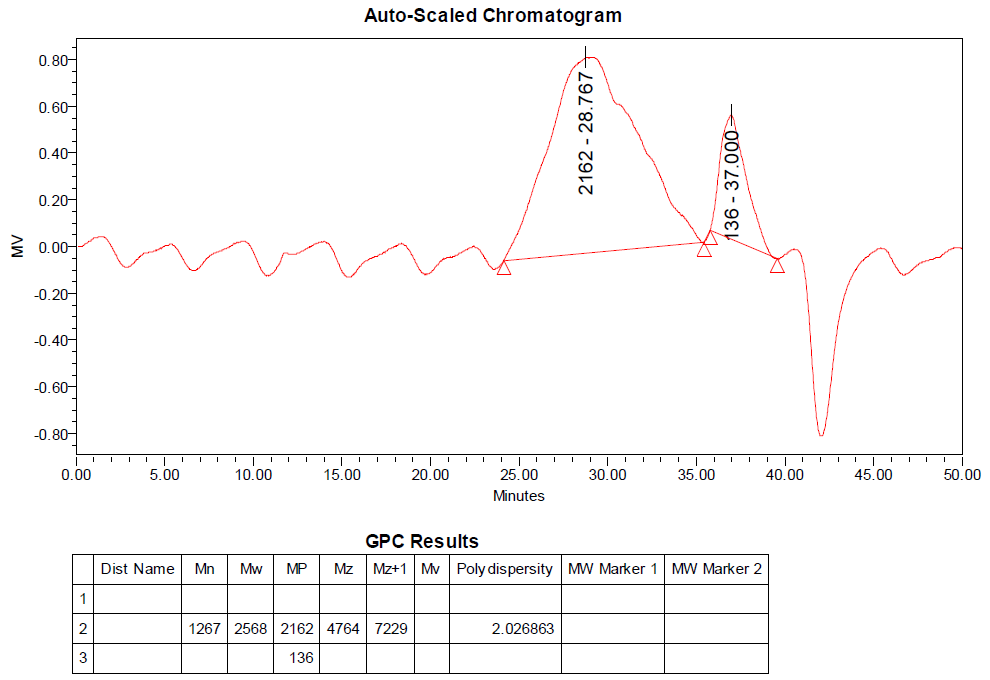


**Figure S12**. GPC and Abs. and Em. of the VySiOSiVycoTerph.


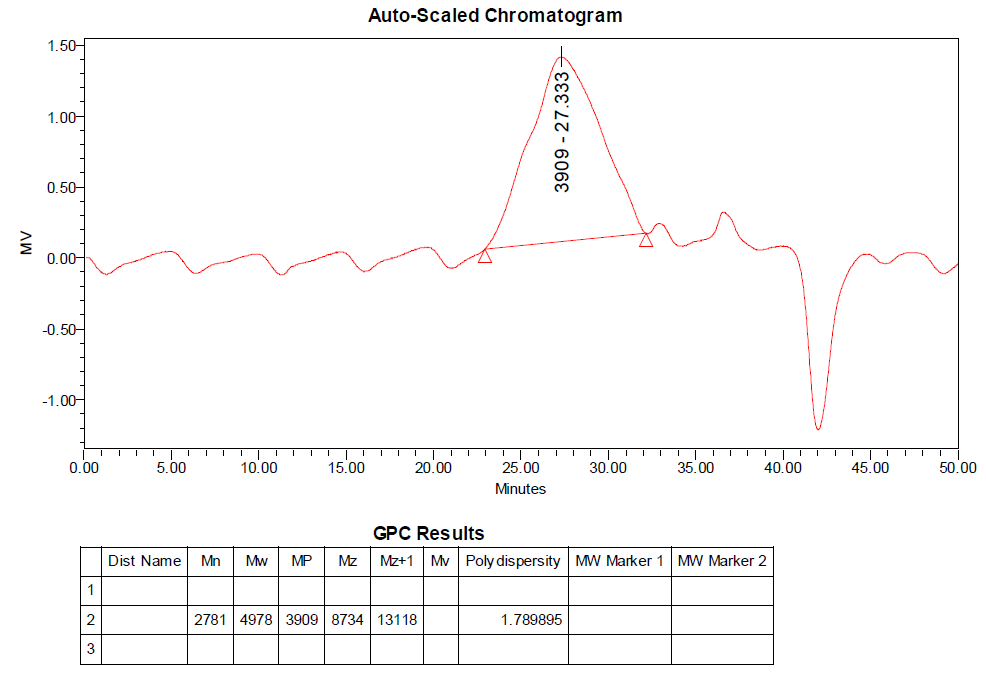


**Figure S13**. GPC of the VySiOSiVycoStil.


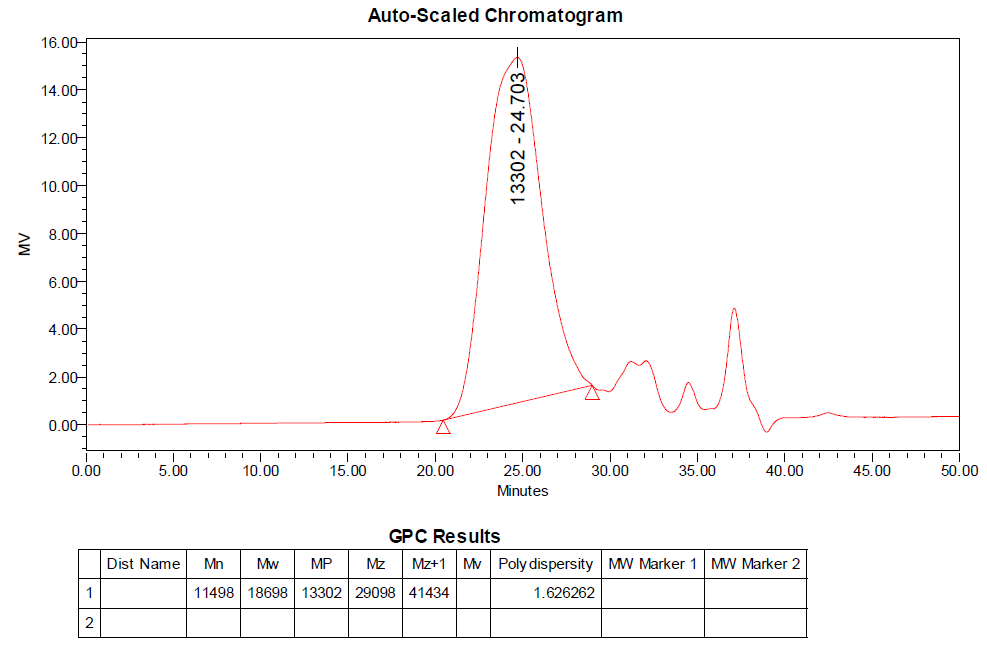


**Figure S14**. GPC of the VySiOSiVycoMe_2_Fl.

**DSC-TGA**

**
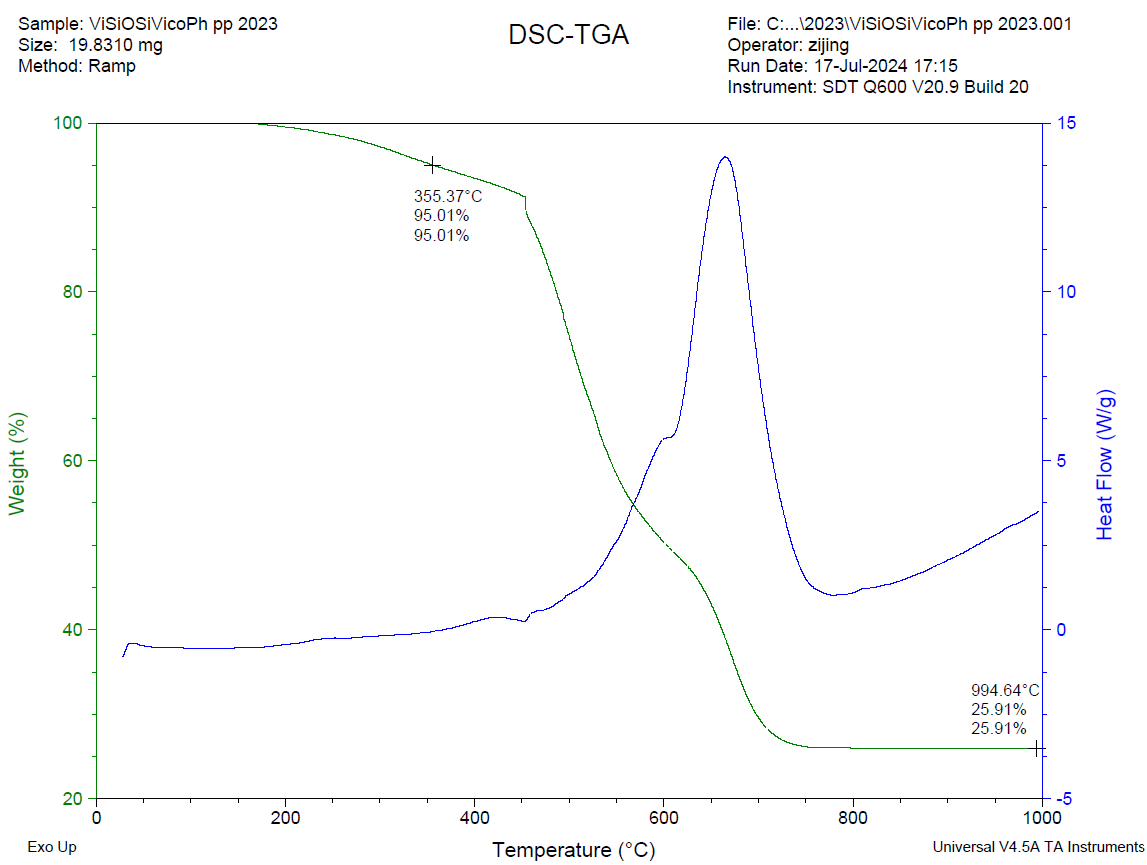
**

**Figure S15**. DSC-TGA of the VySiOSiVycoPh.

**
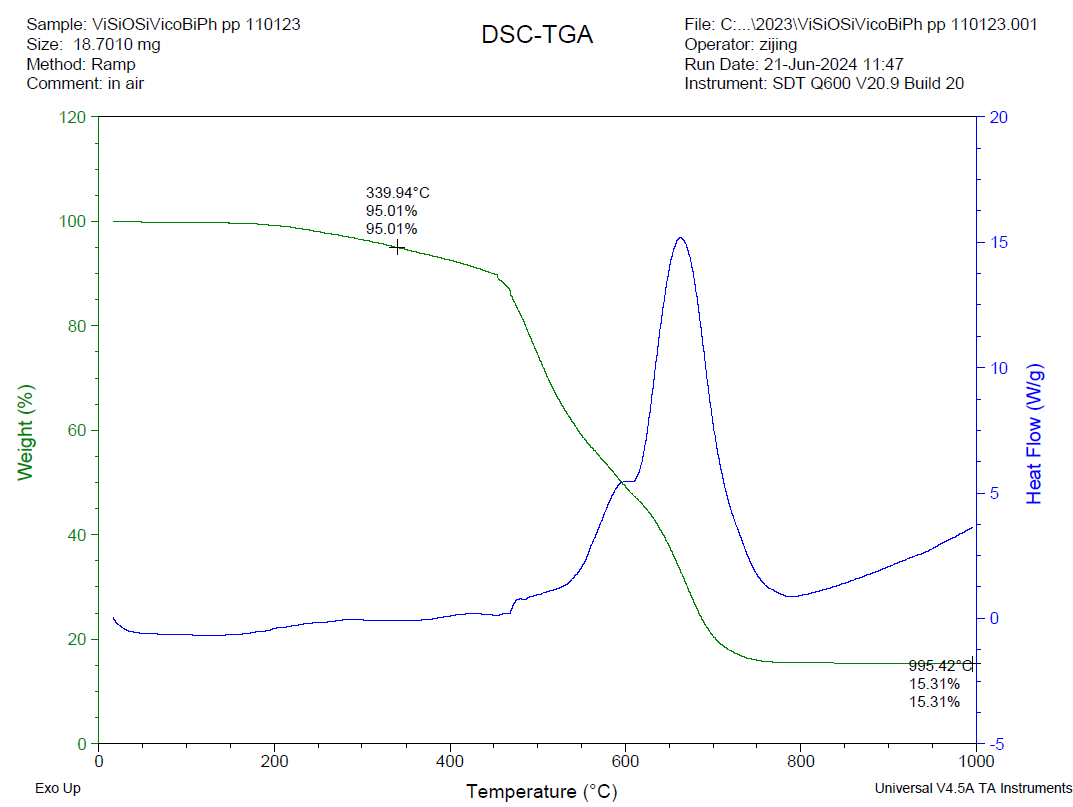
**

**Figure S16**. DSC-TGA of the VySiOSiVycoBiph.

**
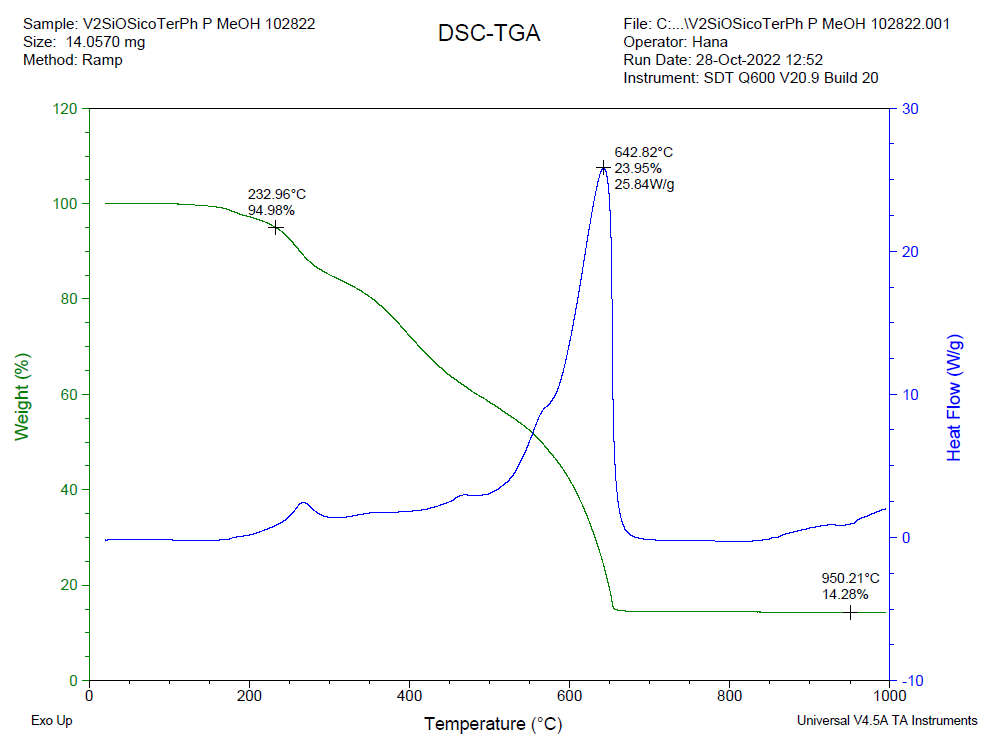
**

**Figure S17**. DSC-TGA of the VySiOSiVycoTerph.

**
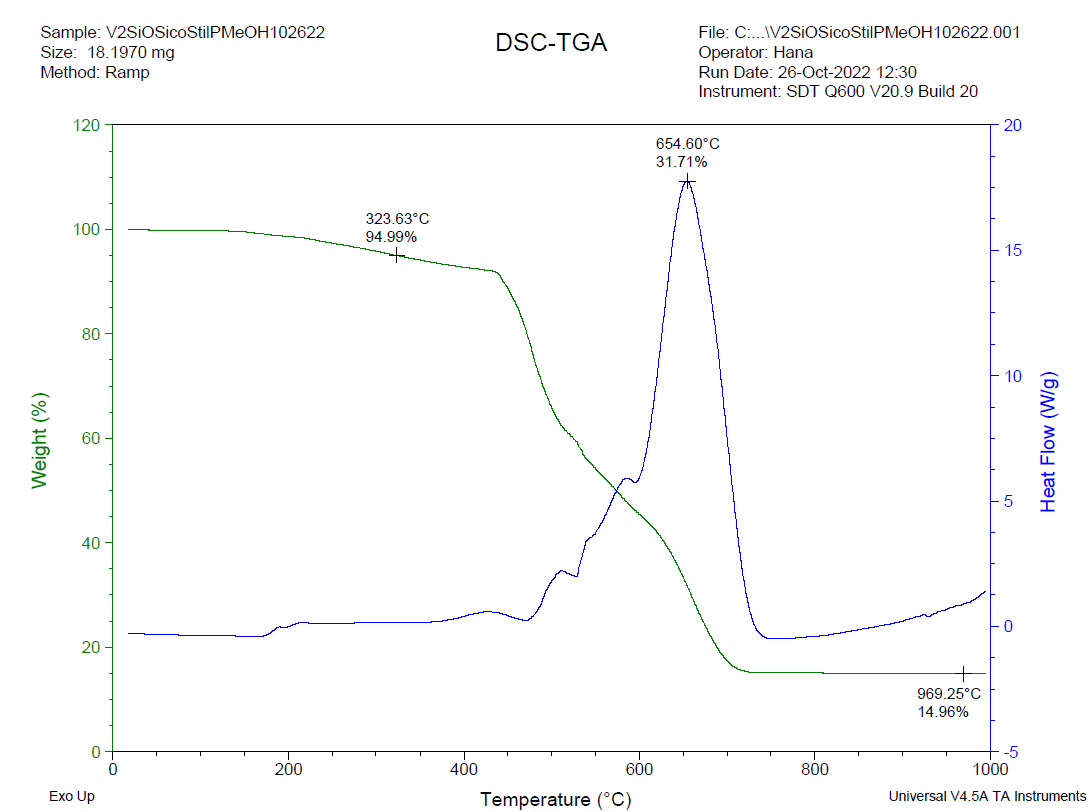
**

**Figure S18**. DSC-TGA of the VySiOSiVycoStil.

**
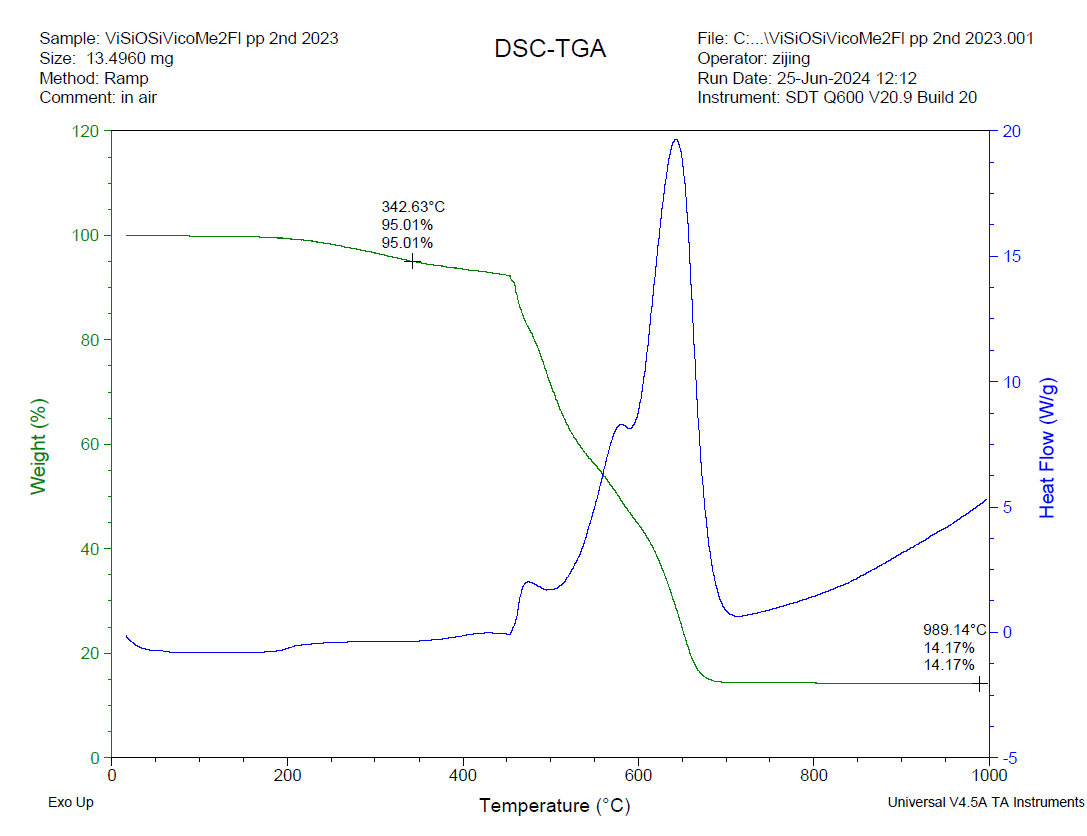
**

**Figure S19**. DSC-TGA of the VySiOSiVycoMe_2_Fl.

**
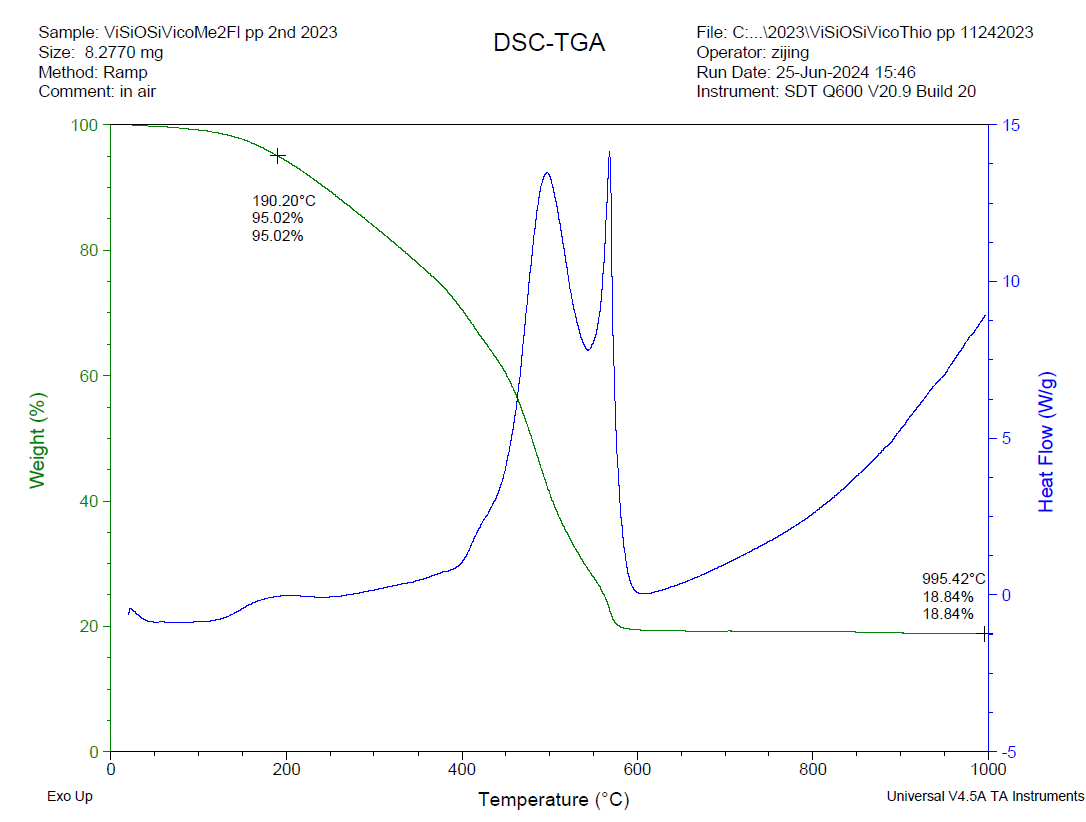
**

**Figure S20**. DSC-TGA of the VySiOSiVycoThio.

**
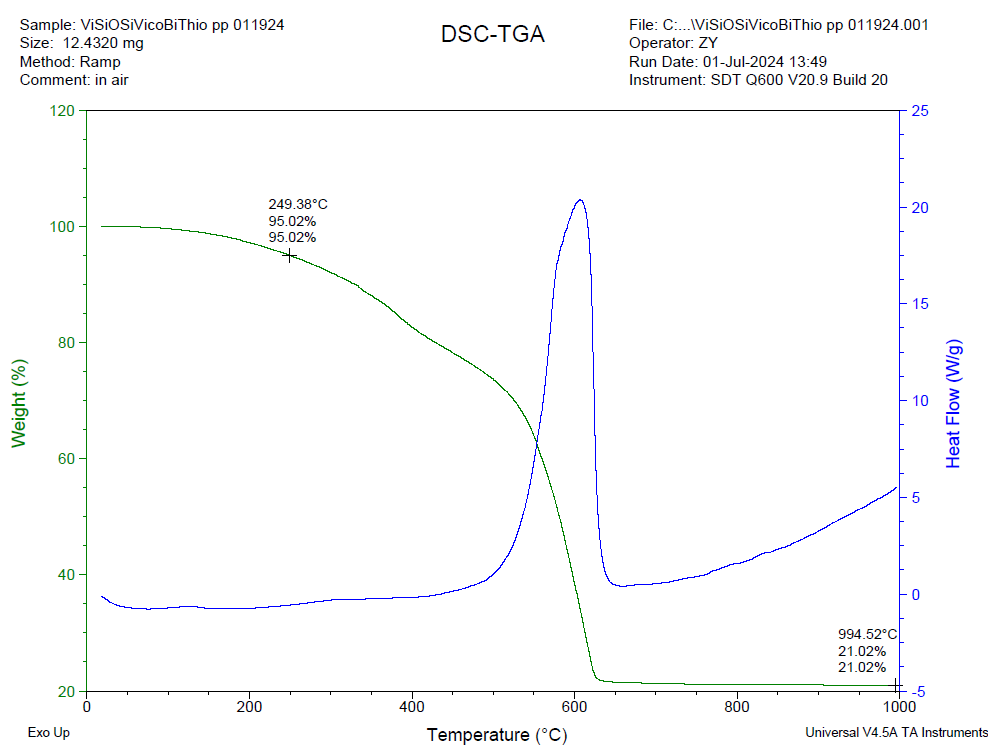
**

**Figure S21**. DSC-TGA of the VySiOSiVycoBithio.

**
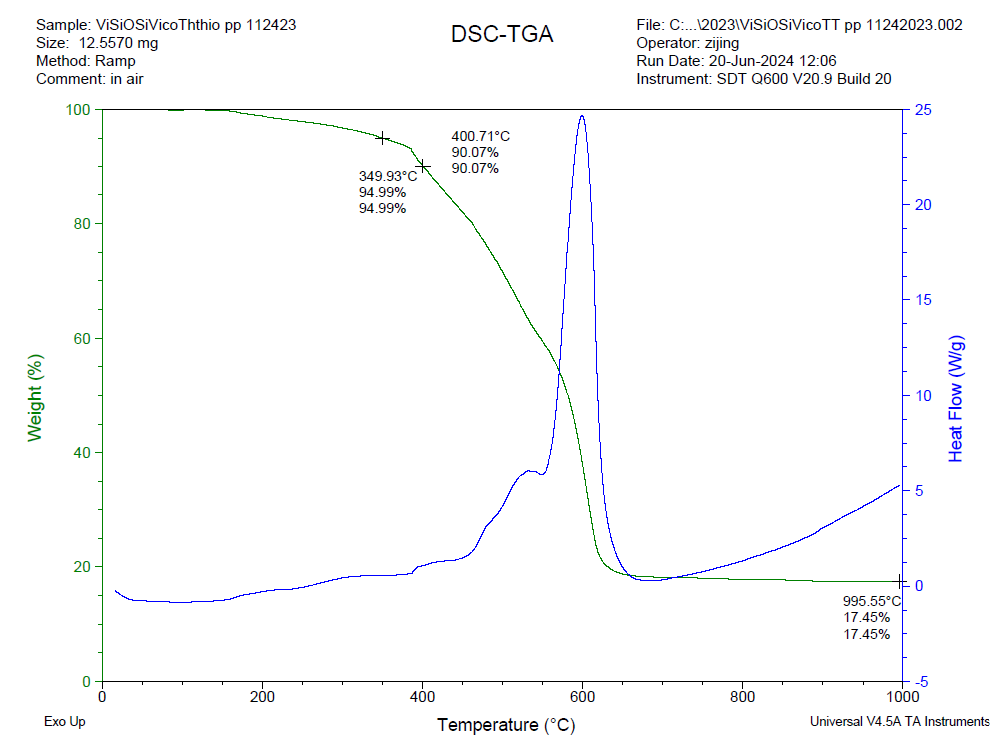
**

**Figure S22**. DSC-TGA of the VySiOSiVycoThthio.


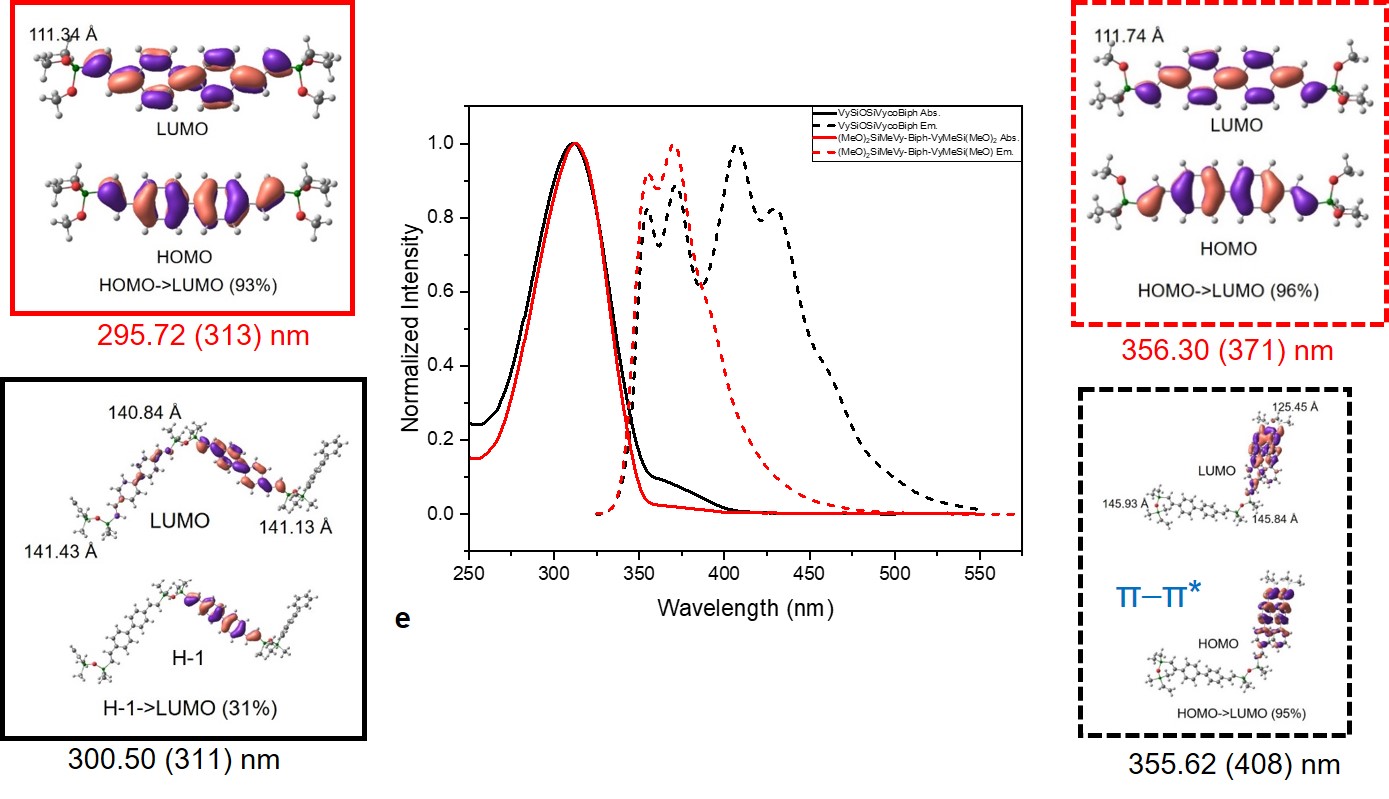


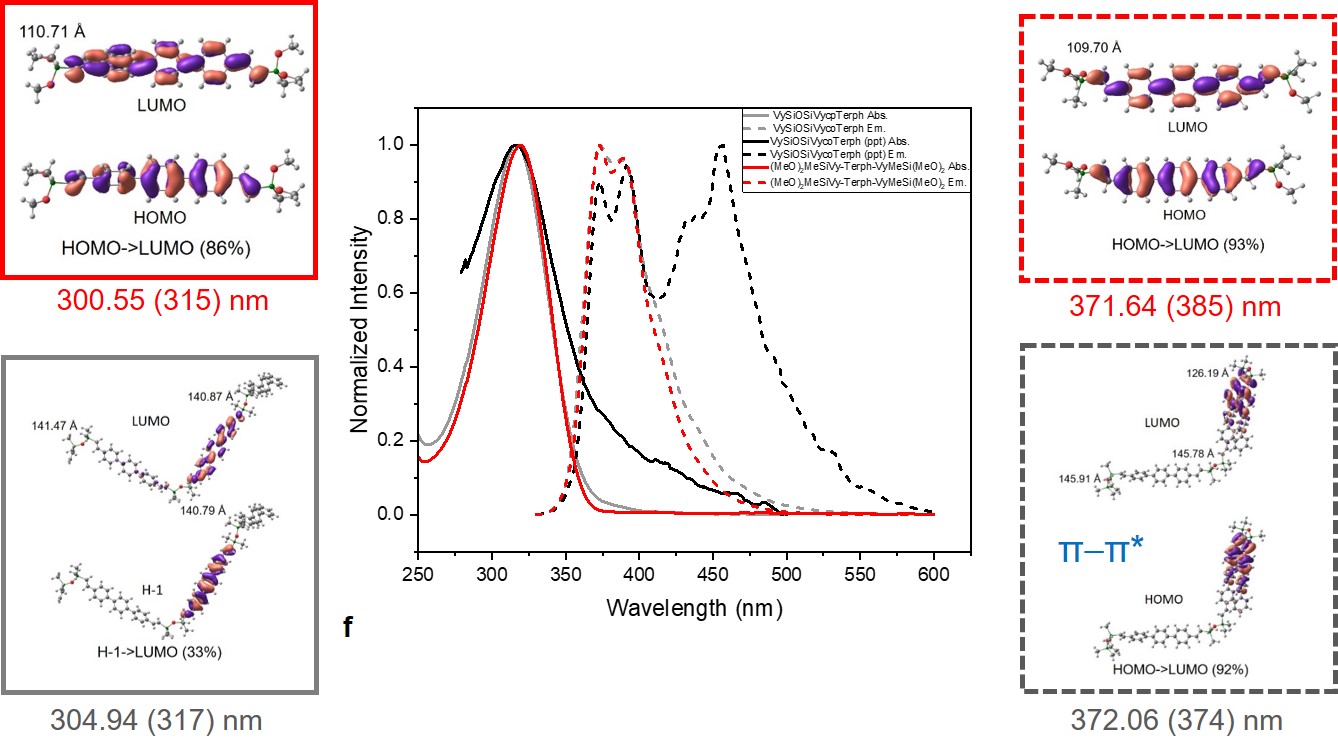


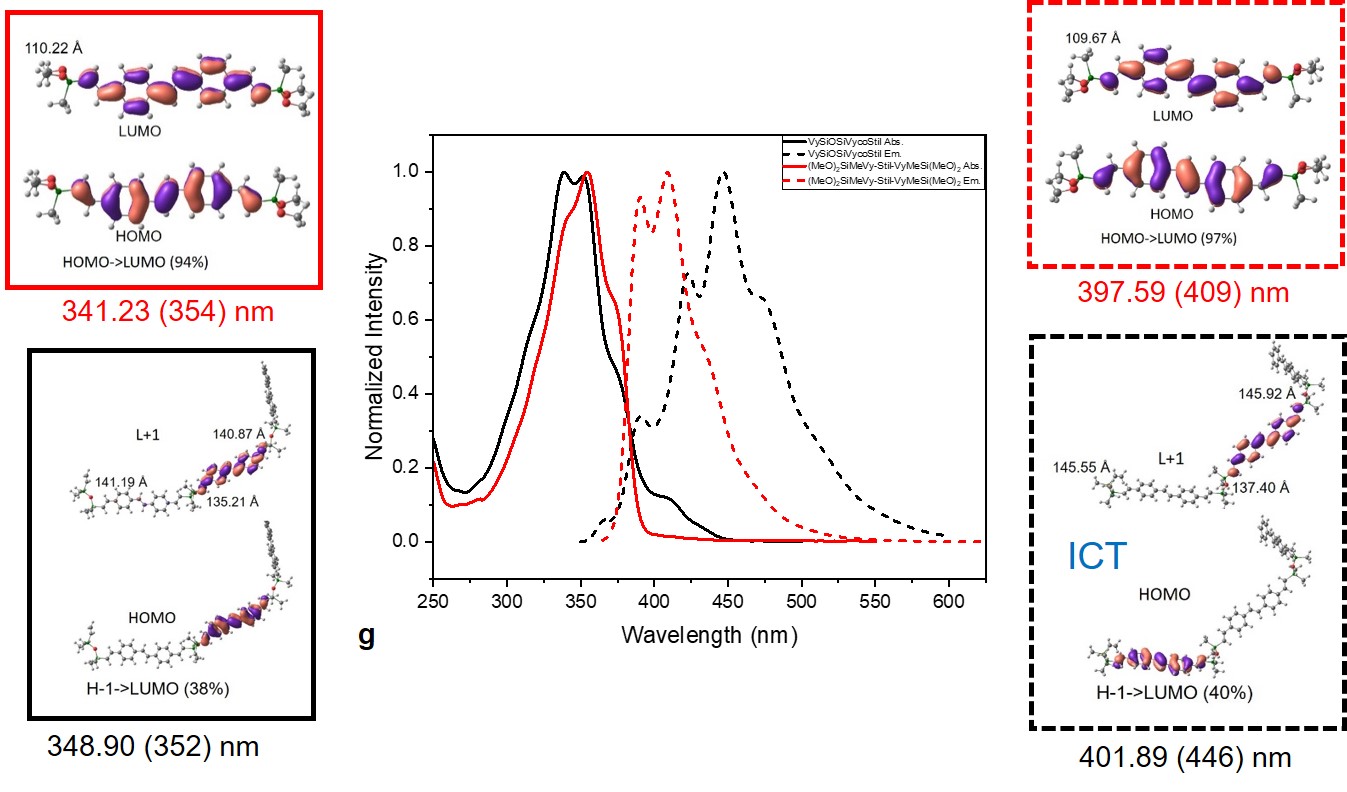


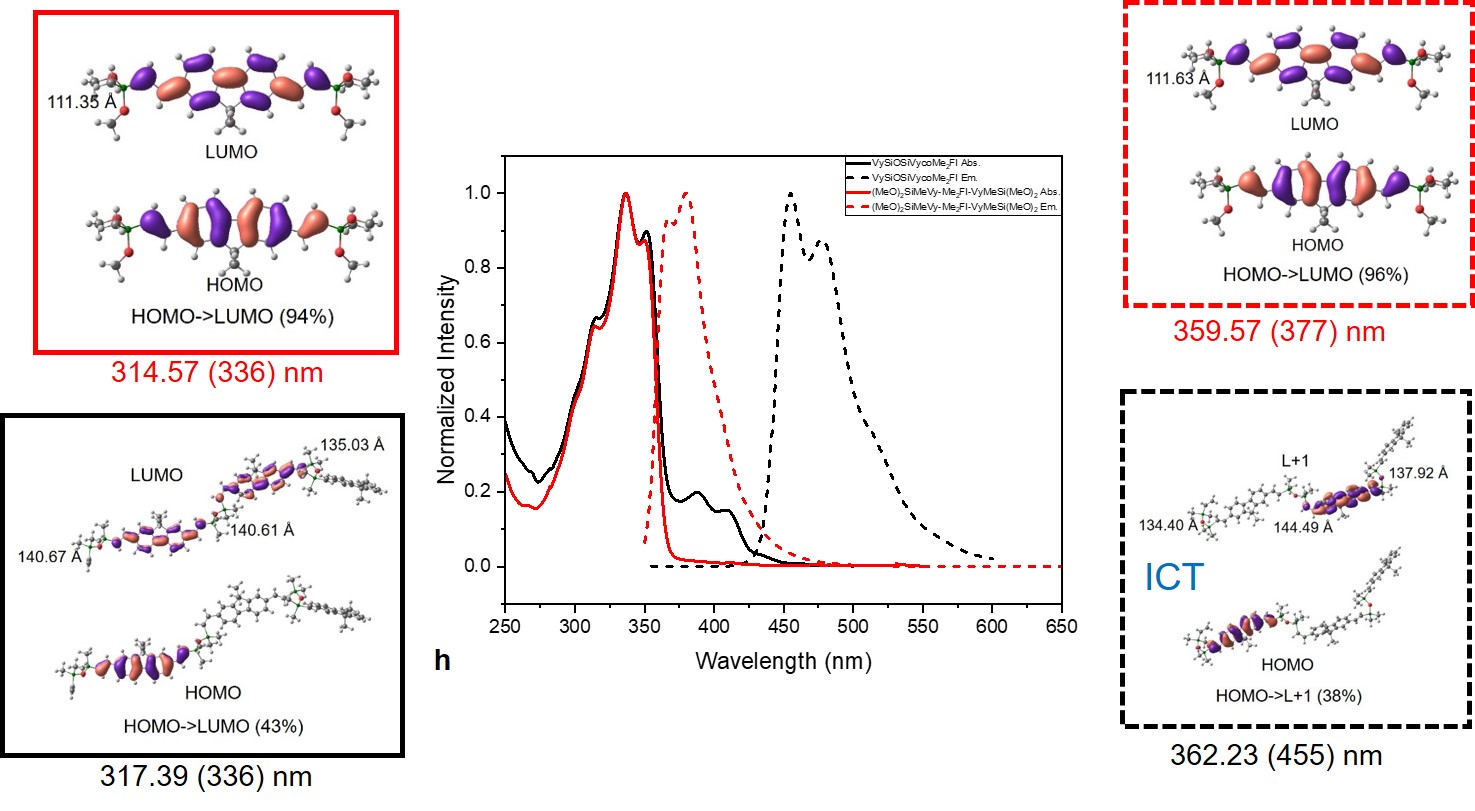


**Figure S23**. Normalized absorption (solid lines) and emission (dashed lines) and corresponding silane model compound found and calculated spectra and structural conformations for **a**. VySiOSiVycoThio (black) and (MeO)_2_SiMeVy-Thio-VyMeSi(OMe)_2_ (red). **b**. for VySiOSiVycoBithio (black) and model compound (MeO)_2_SiMeVy-Bithio-VyMeSi(OMe)_2_ (red). **c**. VySiOSiVycoThthio (black) and (MeO)_2_SiMeVy-Ththio-VyMeSi(OMe)_2_ (red). **d**. VySiOSiVycoPh (black) and (MeO)_2_SiMeVy-Ph-VyMeSi(OMe)_2_ (red). **e**. VySiOSiVycoBiph (black) and (MeO)_2_SiMeVy-Biph-VyMeSi(OMe)_2_ (red). **f**. VySiOSiVycoTerph (ppt) (black) and VySiOSiVycoTerph (grey) and (MeO)_2_SiMeVy-Terph-VyMeSi(OMe)_2_ (red). **g**. VySiOSiVycoStil (black) and (MeO)_2_SiMeVy-Stil-VyMeSi(OMe)_2_ (red). **h**. VySiOSiVycoMe_2_Fl (black) and (MeO)_2_SiMeVy-Me_2_Fl-VySiMe(OMe)_2_ (red).

| **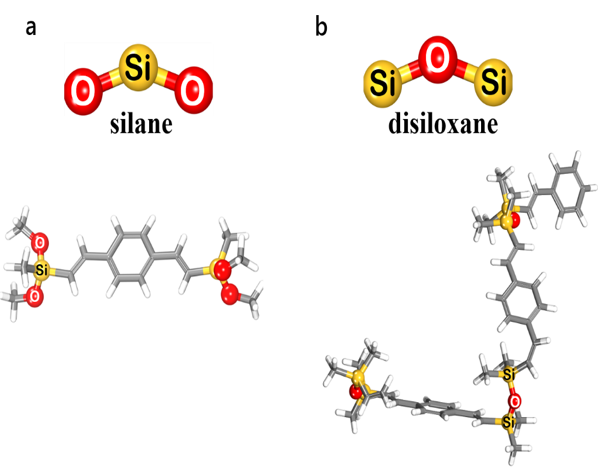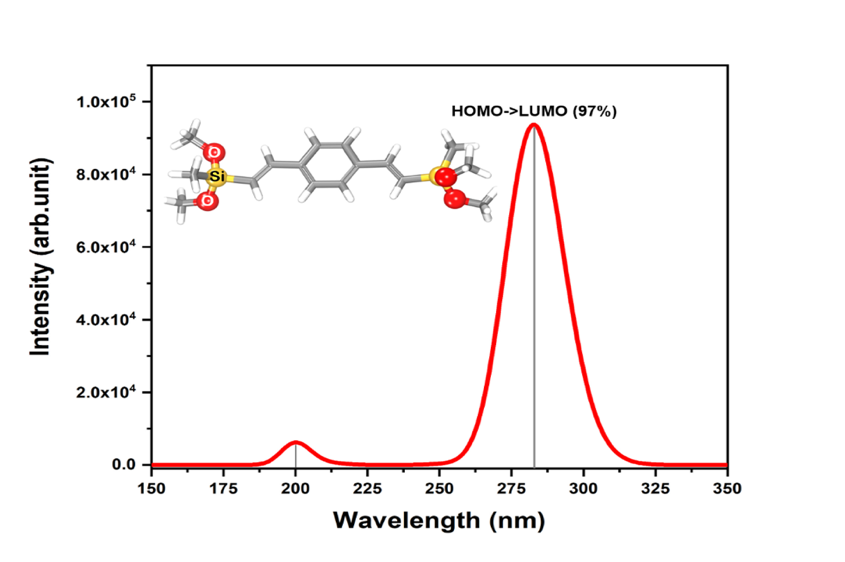**  **Figure S24**. Geometry optimized (MeO)_2_MeSiVy-Ph-VyMeSi(OMe)_2_ structure. **a.** Silane model compound **b.** VySiOSiVycoPh (Disiloxane copolymer) **c**. calculated absorption. |
| --- |
| 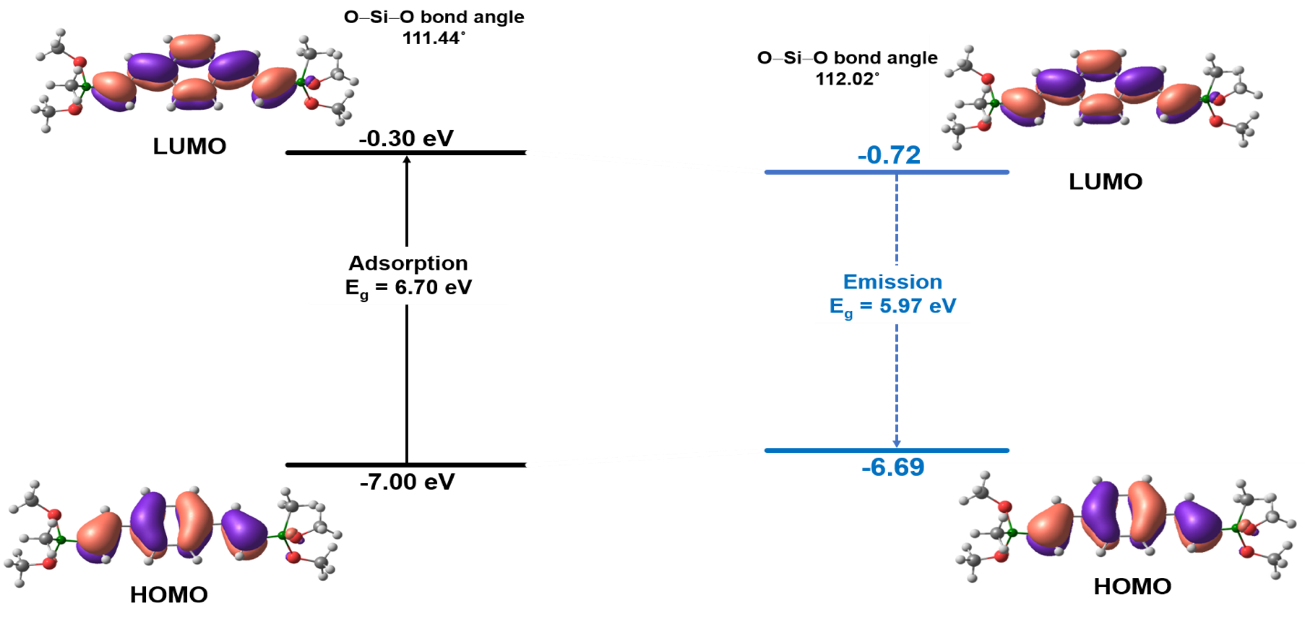 |
| **Figure S25**. HOMO and LUMO of (MeO)_2_SiMeVy-Ph-VyMeSi(OMe)_2_ (Silane model compound) in the absorption and emission states. |

| 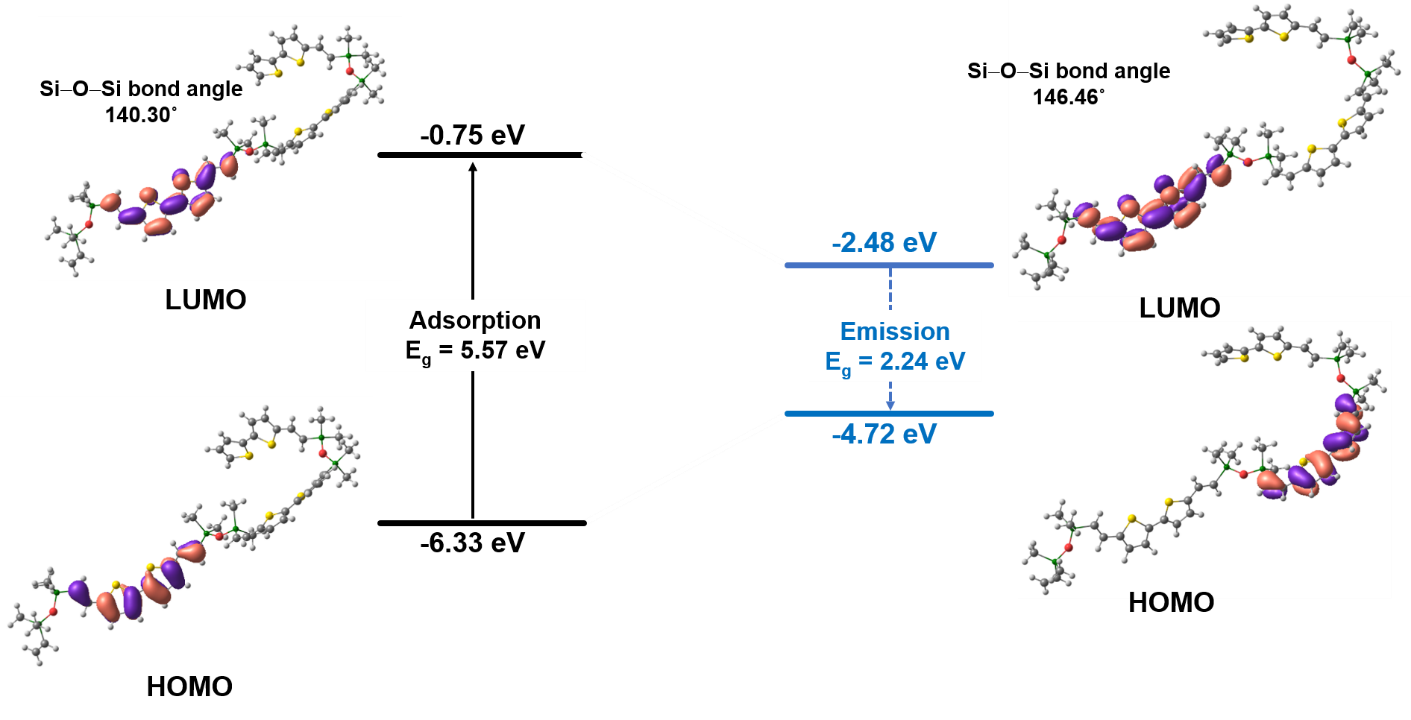 |
| --- |
| **Figure S26**. HOMO and LUMO of VySiOSiVycoBithio (Disiloxane copolymer) at absorption and emission state. |

**Effect of DP**


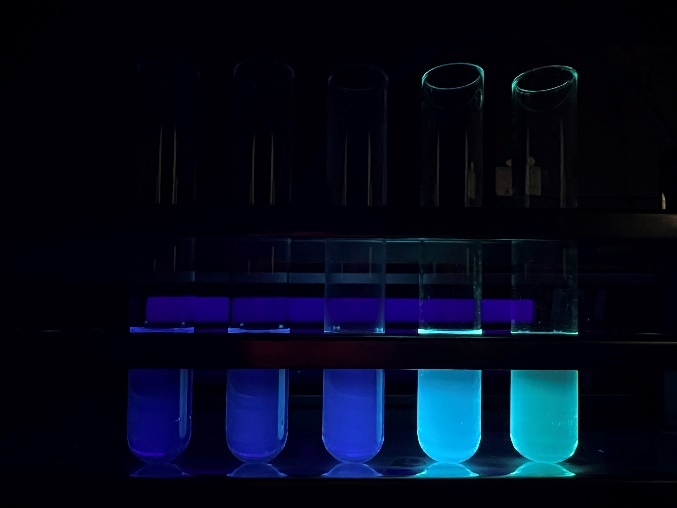


**E**

**F**

**G**

**H**

**I**

**Figure S27**. Fluorescence of VySiOSiVycoStil with different DPs under black light illumination at 365 nm.

**Figure S28**. Normalized GPC curve of VySiOSiVycoStil with different DPs.

**Figure S29**. Normalized UV-vis Absorption and Fluorescence Emission spectra of VySiOSiVycoStil with different DPs.

**Table S4**. UV-vis Absorption, Fluorescence Emission λmax and GPC data of VySiOSiVycoStil with different DPs.

| Samples | Abs. (nm) | Em. (nm) ^§^ | Mw | DP ^†^ |
| --- | --- | --- | --- | --- |
| E | 331 | 378, 397 | 1001 | 2.3 |
| F | 341 | 390, 406 | 1220 | 2.8 |
| G | 342 | 391, 412 | 1431 | 3.2 |
| H | 344 | 391, 409, 443, 470 | 2043 | 4.6 |
| I | 353 | 393, 412, 444, 471 | 7518 | 17.0 |

DP ^†^ is estimated from GPC Mw Em. ^§^ was collected when excited at 352 nm.


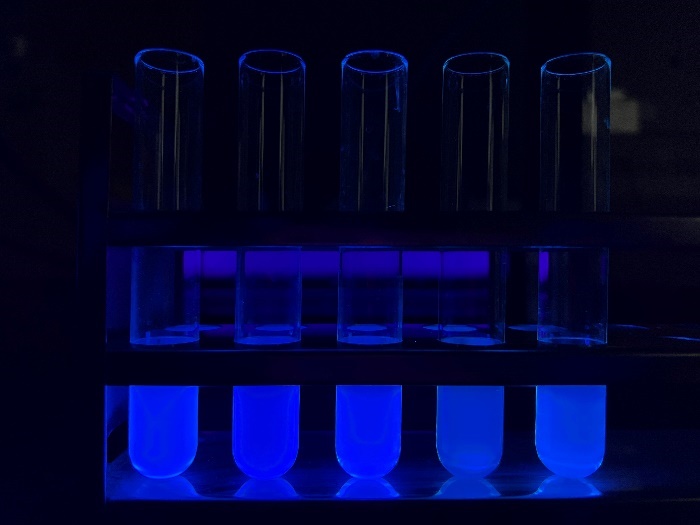


**J**

**K**

**L**

**M**

**N**

**Figure S30**. Fluorescence of VySiOSiVycoBiph with different DP under black light illumination at 365 nm.

**Figure S31**. Normalized GPC curve of VySiOSiVycoBiph with different DP.

**Figure S32**. Normalized UV-vis Absorption and Fluorescence Emission spectra of VySiOSiVycoBiph with different DP.

**Table S5**. UV-vis Absorption, Fluorescence Emission λmax and GPC data of VySiOSiVycoBiph with different DP.

| Samples | Abs. (nm) | Em. (nm) ^§^ | Mw | DP ^†^ |
| --- | --- | --- | --- | --- |
| J | 303 | 354 | 2772 | 6.7 |
| K | 303 | 354, 370 | 2974 | 7.1 |
| L | 307 | 354, 370, 402 | 3099 | 7.4 |
| M | 308 | 354, 372, 404, 426 | 3158 | 7.6 |
| N | 309 | 354, 372, 406, 426 | 3327 | 8.0 |

DP ^†^ is estimated from GPC Mw Em. ^§^ was collected when excited at 305 nm.


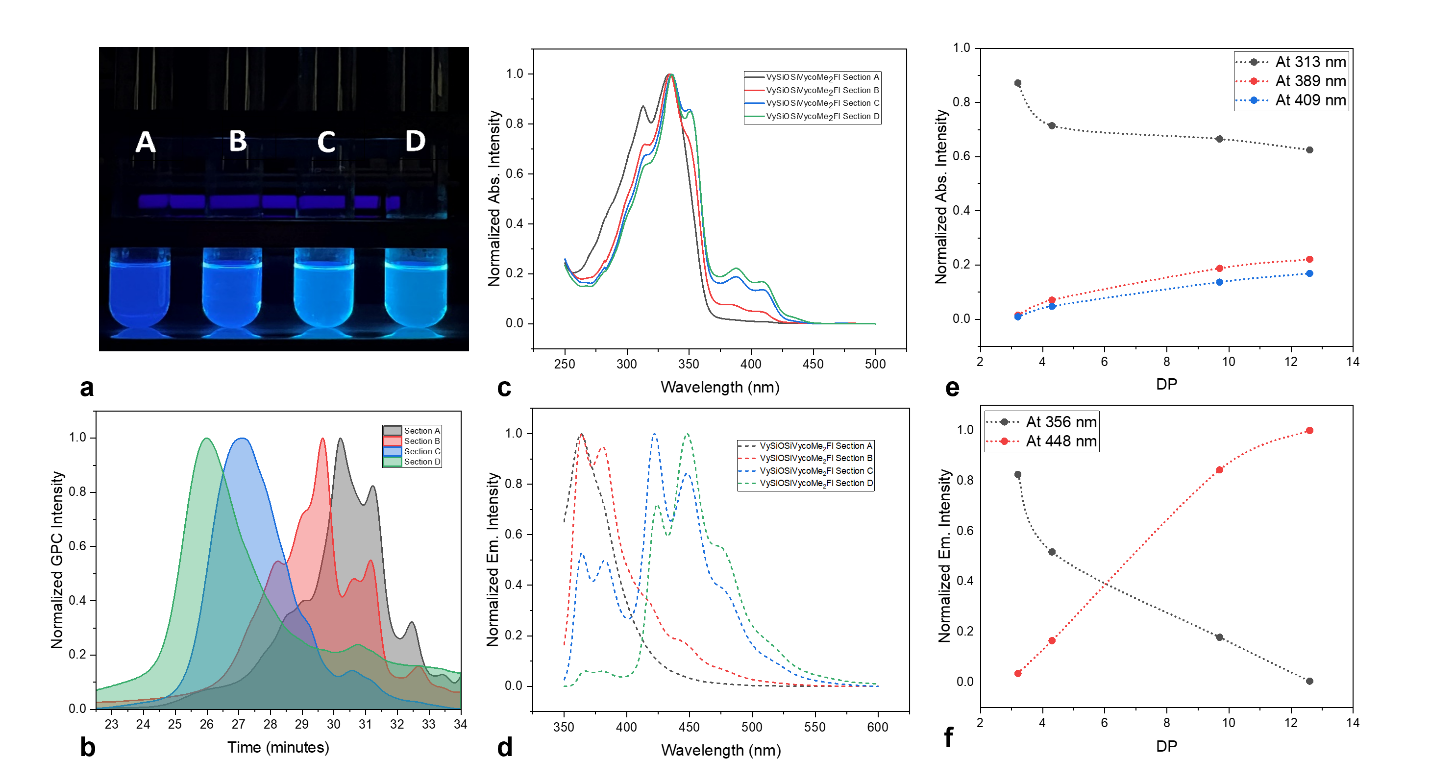


**Figure S33**. **a**. Corresponding fluorescence emission for different DPs associated VySiOSiVycoMe_2_Fl fractions under black light illumination at 365 nm. **b**. GPC traces normalized vs DP for selected VySiOSiVycoMe_2_Fl fractions. **c**. Effect of changes in VySiOSiVycoMe_2_Fl oligomer DPs on normalized UV-vis absorption spectra. **d**. Effect of changes in VySiOSiVycoMe_2_Fl DP on normalized fluorescence emission spectra **e**. GPC determined DPs vs normalized absorption intensities at 313, 389, 409 nm. **f**. GPC determined DPs vs normalized emission λ_max_ at 356 and 448 nm.

**Table S6**. UV-vis Absorption, Emission λ_max_ and GPC data for VySiOSiVycoMe_2_Fl vs DP.

| Sample | Abs. (nm) | Em. (nm) ^§^ | Mw | DP ^†^ |
| --- | --- | --- | --- | --- |
| A | 313, 334 | 364 | 1474 | 3.2 |
| B | 314, 335, 384, 407 | 364, 380 | 1967 | 4.3 |
| C | 336, 350, 388, 410 | 365, 382, 422, 448 | 4431 | 9.7 |
| D | 336, 351, 388, 410 | 367, 382, 424, 447 | 5758 | 12.6 |

DP ^†^ is estimated from GPC Mw ^§^ Em. observed on excitation at 336 nm.

**Charge transfer studies.**


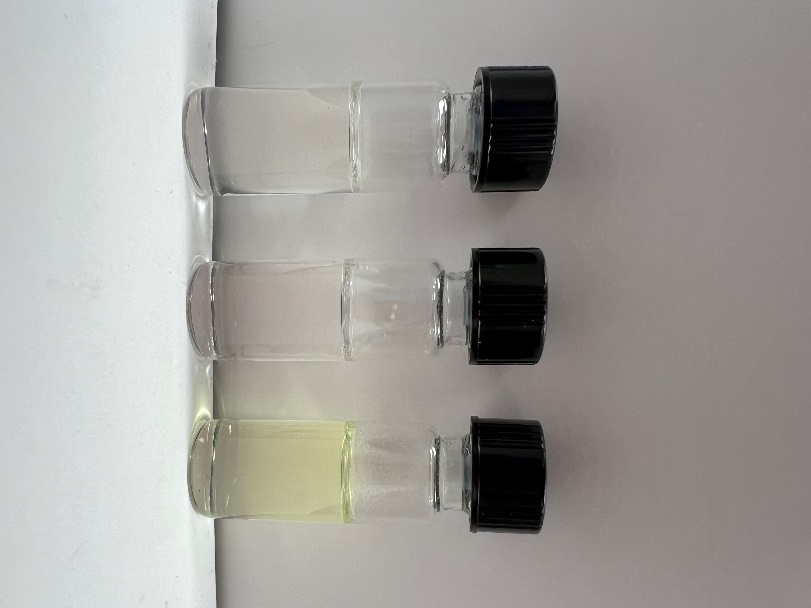


**Figure S34**. VySiOSiVy copolymers before doped with F_4_TCNQ. (from left to right: coBiph, coTerph, coStil)


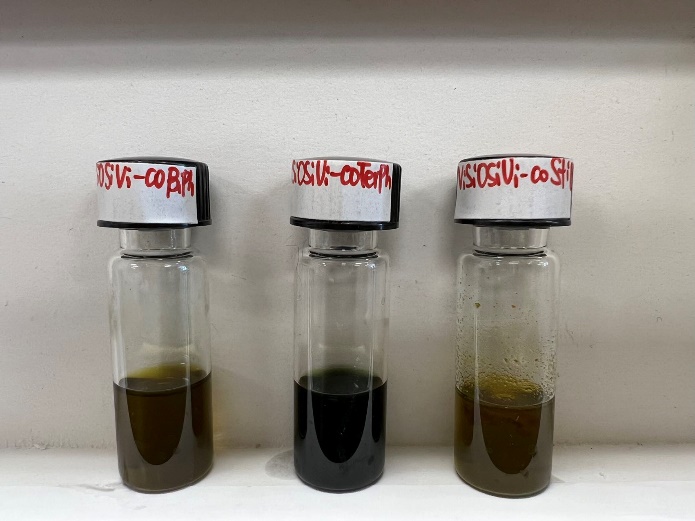


**Figure S35**. VySiOSiVy copolymers doped with 50_mol_% F_4_TCNQ. (from left to right: coBiph, coTerph, coStil)

**Figure S36**. Absorption of VySiOSiVy copolymers doped with 50_mol_% F_4_TCNQ.


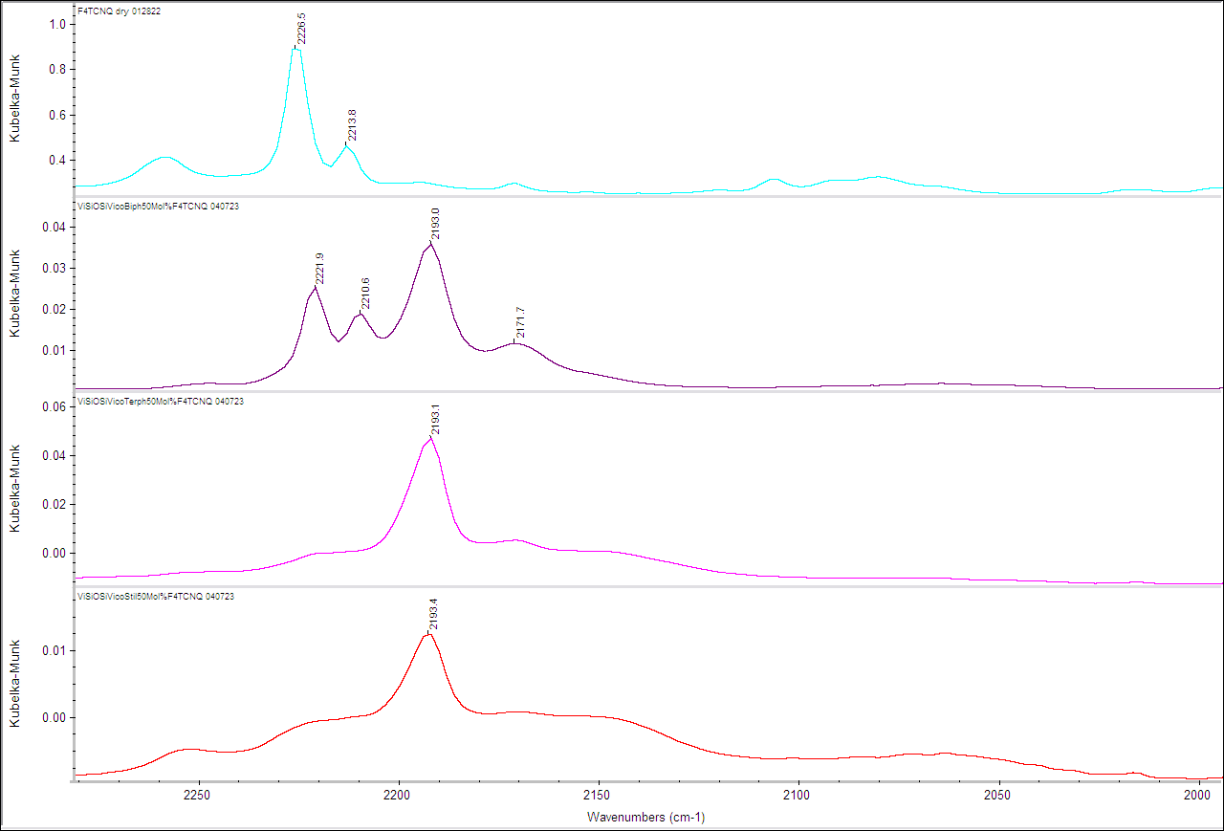


coStil with F_4_TCNQ

coTerph with F_4_TCNQ

coBiph with F_4_TCNQ

F_4_TCNQ

**Figure S37**. FTIR spectra of VySiOSiVy copolymers doped with 50 mol% F_4_TCNQ.

**Table S7**. VySiOSiVycopolyjmers doped with 50_mol_% F_4_TCNQ.

| Copolymers | Mass(mg) | Solvent (ml) (DCM) | F_4_TCNQ | FTIR  (cm^-1^) | Abs (nm) |
| --- | --- | --- | --- | --- | --- |
| VySiOSiVycoBiPh | 10 | 2 | 50 mol% | 2193, 2222, 2211 | 760, 854 |
| VySiOSiVycoTerPh | 10 | 2 | 50 mol% | 2193 | 759, 859 |
| VySiOSiVycoStil | 10 | 2 | 50 mol% | 2193 | 760, 865 |
| F_4_TCNQ |  | - | - | 2227 | 390 |

**Scheme S2**. Syntheses of Vy_4_XDD and Vy_4_HC copolymers.^[1,2]^

|  |  |
| --- | --- |

**Figure S38a**. Φ_F_ and **b**. molar absorptivities of Vy_4_XDD, Vy_4_HC and VySiOSiVy copolymers.

**b**

**a**

**Structure-photophysical property mapping of Vy_4_XDD, Vy_4_HC and VySiOSiVy copolymers**

**Table S8**. Molar absorptivities of Vy_4_XDD, Vy_4_HC and VySiOSiVy copolymers.

| Molar Absorptivity  (10^4^ M⁻¹⋅cm⁻¹) | Vy_4_XDD | Vy_4_HC | VySiOSiVy |
| --- | --- | --- | --- |
| coThio | 1.14±0.15 | 1.78±0.005 | 1.35±0.03 |
| coPh | 4.23±0.41 | 2.81±0.002 | 2.66±0.15 |
| coBiPh | 4.94±0.10 | 4.79±0.06 | 4.11±0.10 |
| coTerPh | 7.24±0.16 | 7.17±0.07 | 2.21±0.07 |
| coStil | 7.40±0.11 | 4.07±0.12 | 2.75±0.005 |
| coBithio | - | - | 1.97±0.02 |
| coThthio | - | - | 1.70±0.05 |
| coMe_2_Fl | - | - | 4.42±0.02 |

**Table S9**. Φ_F_ for Vy_4_XDD, Vy_4_HC and VySiOSiVy copolymers.

| Φ_F_ % | Vy_4_XDD | Vy_4_HC | VySiOSiVy |
| --- | --- | --- | --- |
| coThio | 9±0.2 | 3±0.2 | 5±1 |
| coPh | 89±1 | 86±1 | 10±1 |
| coBiPh | 60±1 | 88±1 | 27±2 |
| coTerPh | 61±1 | 75±1 | 59±7 |
| coStil | 71±3 | 50±3 | 34±4 |
| coBithio | - | - | 23±3 |
| coThthio | - | - | 18±2 |
| coMe_2_Fl | - | - | 78±9 |
